# Electroacupuncture protective effects after cerebral ischemia are mediated through miR-219a inhibition

**DOI:** 10.1186/s40659-023-00448-z

**Published:** 2023-06-30

**Authors:** Yaling Dai, Sinuo Wang, Minguang Yang, Peiyuan Zhuo, Yanyi Ding, Xiaoling Li, Yajun Cao, Xiaoqin Guo, Huawei Lin, Jing Tao, Lidian Chen, Weilin Liu

**Affiliations:** 1 The Institute of Rehabilitation Industry, Fujian University of Traditional Chinese Medicine, Fuzhou, Fujian 350122 China; 2 National-Local Joint Engineering Research Center of Rehabilitation Medicine Technology, Fujian University of Traditional Chinese Medicine, Fuzhou, Fujian 350122 China; 3 Traditional Chinese Medicine Rehabilitation Research Center of State Administration of Traditional Chinese Medicine, Fujian University of Traditional Chinese Medicine, Fuzhou, Fujian 350122 China

**Keywords:** Vascular cognitive impairment, Synaptic plasticity, Neuron, MicroRNA, NMDAR

## Abstract

**Background:**

Electroacupuncture (EA) is a complementary and alternative therapy which has shown protective effects on vascular cognitive impairment (VCI). However, the underlying mechanisms are not entirely understood.

**Methods:**

Rat models of VCI were established with cerebral ischemia using occlusion of the middle cerebral artery or bilateral common carotid artery. The brain structure and function imaging were measured through animal MRI. miRNA expression was detected by chip and qPCR. Synaptic functional plasticity was detected using electrophysiological techniques.

**Results:**

This study demonstrated the enhancement of Regional Homogeneity (ReHo) activity of blood oxygen level-dependent (BOLD) signal in the entorhinal cortical (EC) and hippocampus (HIP) in response to EA treatment. miR-219a was selected and confirmed to be elevated in HIP and EC in VCI but decreased after EA. N-methyl-D-aspartic acid receptor1 (NMDAR1) was identified as the target gene of miR-219a. miR-219a regulated NMDAR-mediated autaptic currents, spontaneous excitatory postsynaptic currents (sEPSC), and long-term potentiation (LTP) of the EC-HIP CA1 circuit influencing synaptic plasticity. EA was able to inhibit miR-219a, enhancing synaptic plasticity of the EC-HIP CA1 circuit and increasing expression of NMDAR1 while promoting the phosphorylation of downstream calcium/calmodulin-dependent protein kinase II (CaMKII), improving overall learning and memory in VCI rat models.

**Conclusion:**

Inhibition of miR-219a ameliorates VCI by regulating NMDAR-mediated synaptic plasticity in animal models of cerebral ischemia.

**Supplementary Information:**

The online version contains supplementary material available at 10.1186/s40659-023-00448-z.

## Introduction

Vascular cognitive impairment (VCI) is defined as clinical stroke or subclinical vascular damage caused by cerebrovascular disease. It is a clinical syndrome involving the impairment of at least one cognitive domain [1], encompassing all cognitive disorders associated with cerebrovascular disease, and may present as a number of symptoms, from mild cognitive impairment to dementia [2]. Pharmacological therapies are in development, and no drug has been approved for the treatment of VCI. Various pharmacological compounds, including donepezil, galantamine, and memantine, which are approved for the treatment of Alzheimer’s disease (AD), have demonstrated modest cognitive benefits in patients affected by VCI [3]. However, the functional benefits of these treatments are inconsistent and their side effects are damaging. Because of these limitations, non-pharmacological therapies are being considered as potential treatments to reduce cognitive dysfunction [4]. The American Academy of Neurology recommends regular nonpharmacologic therapies to improve cognitive functions [5]. Research has focused on non-pharmacological therapies aimed at activating endogenous neural repair pathways to improve cognitive impairments. Studies have indicated that electroacupuncture (EA) may reduce cell death and improve neural function recovery [6]. It is recognized as a superior therapeutic option for VCI as a nonpharmacological therapy in traditional Chinese medicine. However, the underlying mechanism has not been fully elucidated.

microRNAs (miRNAs) are molecules that epigenetically regulate gene expression [7]. miRNAs not only play important roles in brain tissue development, neuron differentiation, and advanced nerve function but also regulate local protein synthesis at synapses involved in learning and memory [8]. Specific inhibition or overexpression of miRNAs may be an important method to influence VCI. For example, inhibition of miR-96 can affect cognitive function in VCI rats [9]. Additionally, overexpression of miR-26b significantly attenuates inflammation and microglial activation, neurotoxicity, and cognitive impairment caused by cerebral ischemia [10].

Specific miRNAs are necessary for N-methyl-D-aspartic acid receptor (NMDAR)-dependent synaptic plasticity through modulation of translation of proteins involved in dendritic spine morphogenesis or synaptic transmission [11]. For example, miR-34a is implicated in cell proliferation, morphology, and function of developing neurons that enhance behavioral functions by inhibiting the density of NMDA-evoked currents [12]. Recent studies have evaluated the significance of local changes in miR-219 and NMDAR expression levels throughout LTP and epilepsy [13]. Therefore, miRNA-related therapies present strategies for alleviating synaptic pathology and cognitive impairment.

Recently, miRNAs have become important to the effects of EA. In particular, EA can regulate the expression of miR-93 and miR-124 to promote nerve regeneration and brain repair to reduce cognitive dysfunction [14]. EA also regulates miRNAs which target synaptic proteins, increasing the number of dendritic spines and synaptic structure in hippocampus [15]. Therefore, EA may possibly play a role in regulating the expression of specific miRNAs involved in synaptic plasticity.

As an endogenously-activated neural repair method, EA has been shown to promote nerve regeneration and repair, improving neurological function after cerebral ischemia [16]. Our study revealed that EA could regulate miR-219a to alleviate VCI by modulating NMDAR-mediated synaptic plasticity in the entorhinal cortical (EC)-hippocampus (HIP) CA1 neural circuit in animal models of cerebral ischemia. Additionally, regulating expression of miR-219a increased NMDAR1 and promoted the phosphorylation of CaMKII, which ultimately improved learning and memory in VCI rat models. These findings provided a theoretical basis for the treatment of VCI with EA.

## Results

### EA can improve the vascular cognitive function of rats with Middle cerebral artery occlusion (MCAO)

Neurological deficits were evaluated to determine a neurological score. Neurological deficits were scored in pre-treatment, and at day 1, 7, and 14 after EA treatment. The results are shown in Fig. S1 C-F. The neurological deficit scores showed no differences in pre-treatment and 24-hours post-treatment in the MCAO group, the EA group, and the Non-acu group (Fig. S1 C-D, *P*>0.05), while after day 7 and 14 post-EA treatment, the MCAO group showed pronounced neurological deficits (Fig. S1 E-F, *P*<0.05).

The learning and memory abilities of rats was evaluated using a step-down test. During the trial period of the step-down test, the latency of the MCAO group was significantly shortened compared to the sham group, while the latency of the EA group was increased (*P*<0.05). There was no statistical difference between the non-acu group and the MCAO group (Fig. S1 H, *P*>0.05).

The results indicated that the escape latency of rats in the sham group was reduced by 70–80% after four days of training, demonstrating that the rats had learned to find the target platform. The MCAO group had a significant increase in escape latency when compared to the sham group (Fig. S1 I, *P*<0.01), while the platform crossing times and the percentage of target quadrant in the memory ability test decreased (Fig. S1 J, *P*<0.05). After EA treatment the escape latency of the MCAO group decreased on day 3–4 (Fig. S1I, P<0.01), while the number of crossing platforms and the percentage of target quadrant increased (Fig. S1K, *P*<0.05).

**Fig. 1:**
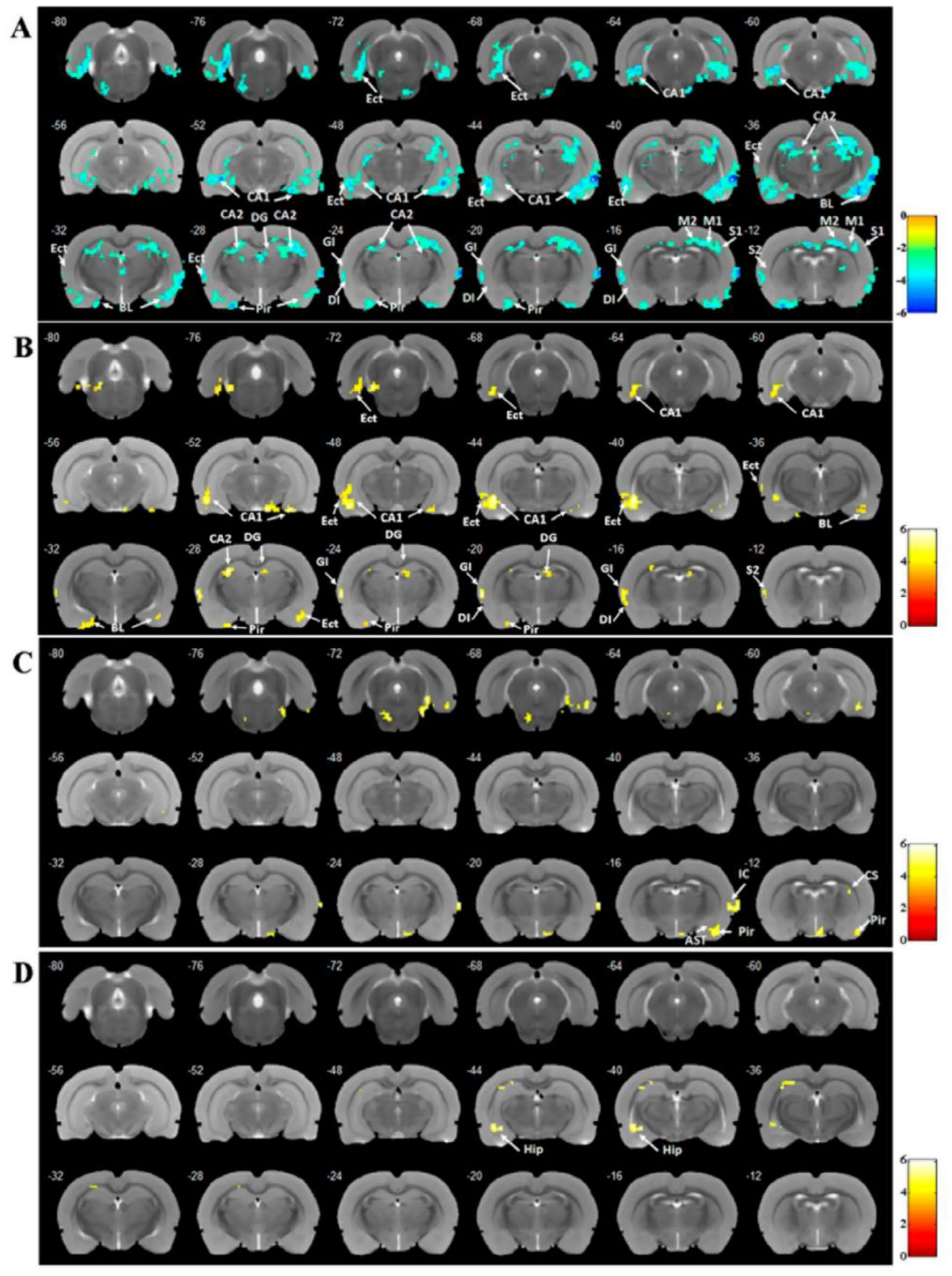
EA could improve pre-to-post changes in ReHo activities of BOLD signals. (**A**) The ReHo activities of BOLD signal of brain region changes in the MCAO group compared with the sham group. (**B**) The ReHo activity changes in the EA group compared with the MCAO group. (**C**) The ReHo activity changes in the Non-acu group compared with the MCAO group. (**D**) The ReHo activity changes in the Non-acu group compared with the EA group. (EC: Entorhinal cortex; CA1: Hippocampus CA1, CA2: Hippocampus CA2; BLA: Basolateral nucleus of the amygdala; DG: Dentate gyrus; M1: Primary motor cortex; M2: Secondary motor cortex.) Brain region were considered significant at *P* < 0.005 and clusters ≥ 20 voxels. (A-D: One-way ANOVA, followed by Tukey’s post hoc test, Data represent the mean ± SD. n = 18 rats/group)

### EA decreased the volume of cerebral infarction in rats with MCAO

After 14 days of treatment, T2-weighted image (T2WI)structural imaging was used to evaluate the cerebral infarction of rats from each group, including HIP, EC, motor cortex, sensory cortex, dorsal thalamus, and striatum regions. The larger the gray area in the image was, the higher the infarct effects were in that particular brain region (Fig. S2A-C). EA treatment reduced cerebral infarct volumes in comparison to the MCAO group (Fig. S2B). There was no statistical difference in cerebral infarct volumes of the non-acupoint group (Fig. S2C, *P*<0.05).

### EA regulated the regional homogeneity (ReHo) activity of blood oxygenation level dependent (BOLD) signals in brain regions of rats with MCAO

After 14 days of treatment, we measured the ReHo activity of BOLD signals across different brain regions of rats in each group (Fig. 1A-D). The MCAO group had a reduced ReHo activity in BOLD signals in the bilateral amygdala, bilateral HIP, bilateral EC, bilateral piriform cortex, bilateral sensory cortex, bilateral motor cortex, bilateral cingulate gyrus, bilateral insular cortex, bilateral dorsal thalamus, left auditory cortex, and left striatum (Fig. 1A). After 14 days of EA treatment, the brain regions with increased ReHo activity of BOLD signal in the EA group included bilateral amygdala, bilateral HIP, bilateral EC, right piriform cortex, right insular cortex, and right sensory cortex (Fig. 1B). In the non-acu group, the left amygdala, the left piriform cortex, and the left striatum were found to have increased BOLD signals (Fig. 1C). Compared to the non-acu group, the ReHo activity of BOLD signals increased in the right hippocampus in the EA group (Fig. 1D). The results for each group of ReHo values are available in the supplemental material (Table S1).

### EA enhanced the synaptic plasticity of EC-HIP CA1 neural circuits in rats with MCAO

**Fig. 2:**
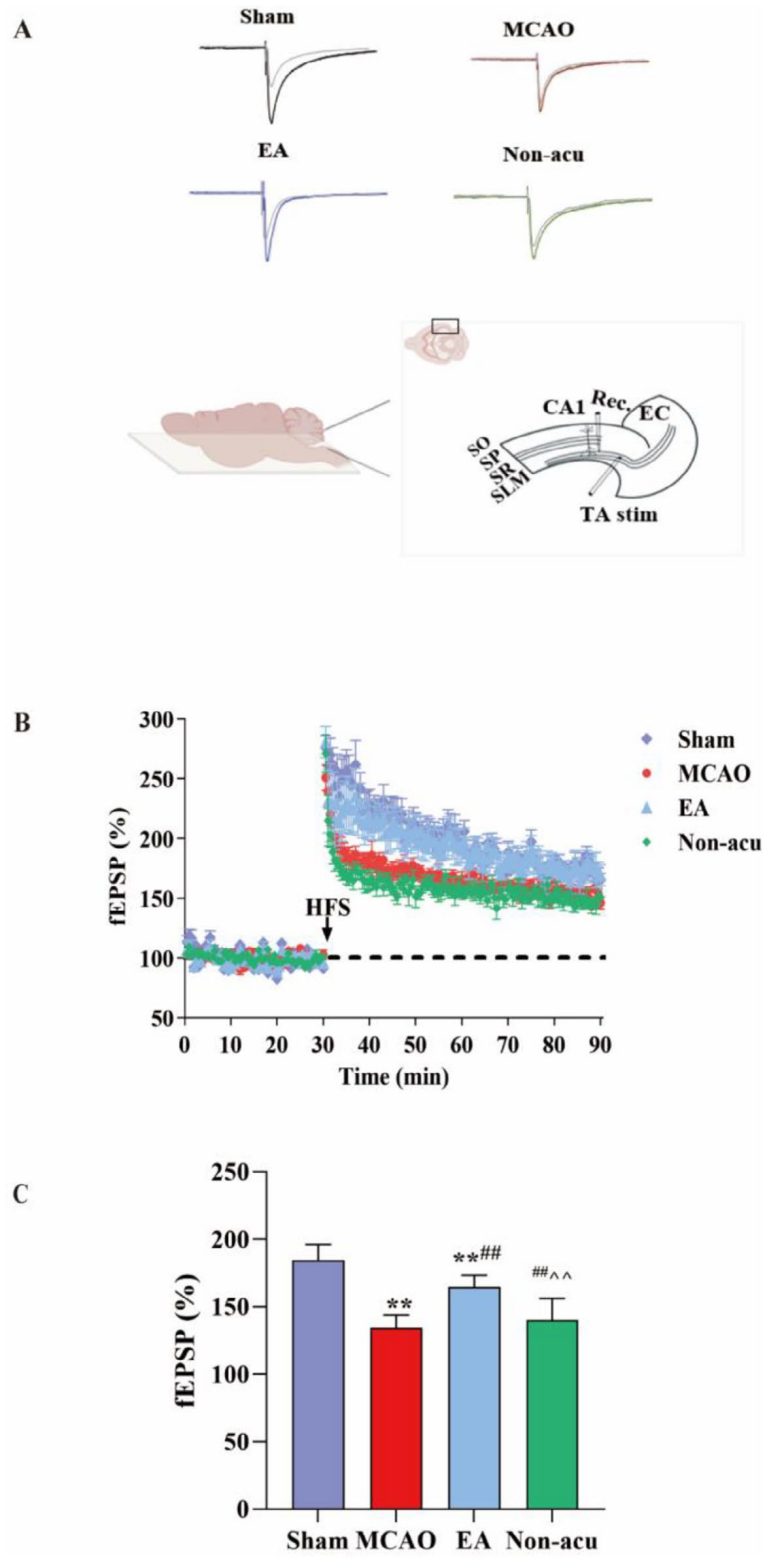
EA may improve synaptic plasticity in MCAO rats (**A**) Schematic representation of electrical stimulation protocols that induce LTP at the EC-CA1 circuit in MCAO rats (**B***B*) Baseline recordings were taken 30 and 60 min after LTP induction. (**C**) Bar graphs show the percentage change in LTP at 50 to 60 min after HFS. *Sham vs. MCAO, #. MCAO vs. EA, ^. EA vs. Non-acu. *P*<0.05.(C. One-way ANOVA, followed by Tukey’s post hoc test, Data represent the mean ± SD. n = 6 sections from 3 rats/group)

High-frequency stimulation (HFS) of the Schaffer collateral inputs to the EC and HIP CA1 neural circuits induced stable LTP in the slope of field excitatory postsynaptic potential (fEPSP) in the Sham group. In this experiment, the degree of LTP was measured and expressed as a percentage of the fEPSP slope (Fig. 2A). The results of these experiments indicated that in the MCAO group, after induction of HFS, the percentage of fEPSP slope of the EC-HIP CA1 circuit on the ischemic side decreased (Fig. 2B, P<0.05). After EA treatment, the percentage of fEPSP slope of EC-HIP CA1 circuit on the ischemic side increased (Fig. 2C, P<0.05). Specifically, the increase in the fEPSP slope percentage indicated that the EA treatment may have led to an enhancement of the LTP response in this circuit.

### MiRNA profiles of entorhinal cortex and hippocampal CA1 were identified in rats with MCAO after EA treatment

**Fig. 3:**
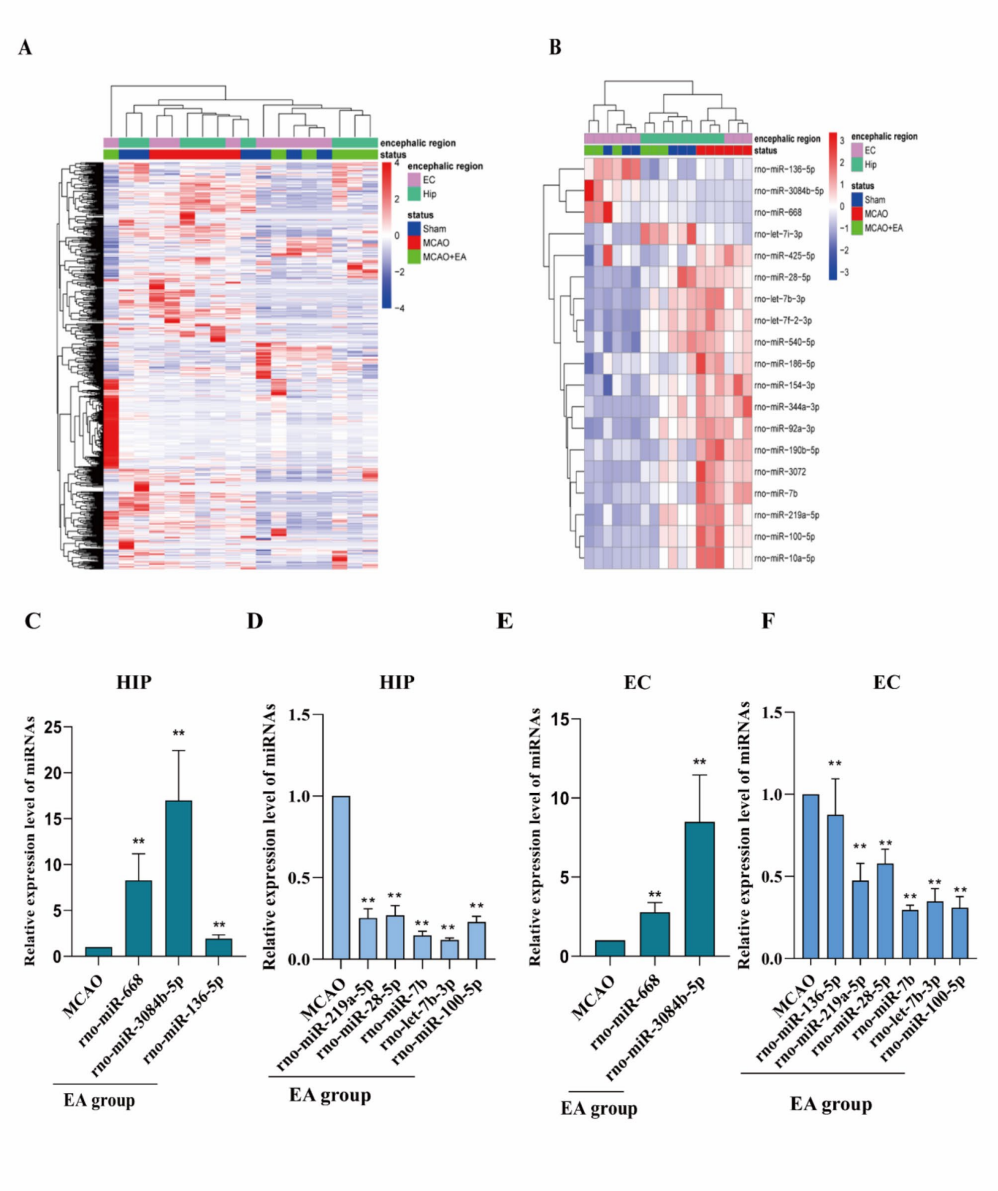
Effect of EA on the expression of CA1 and miRNAs in the ischemic hippocampus and entorhinal cortex of MCAO rats (**A**) Total RNA extracted from hippocampus and entorhinal cortex in ischemic areas for miR microarray analysis. (**B**) The changes in miRNA levels in both hippocampal CA1, and in the EC of the ischemic side. (**C**) Up-regulated miRNA in the hippocampus. (**D**) Down-regulated miRNA in the hippocampus. (**E**) Up-regulated miRNA in the entorhinal cortex. (**F**) Down-regulated miRNA in the entorhinal cortex.*. Sham vs. MCAO, #. MCAO vs. EA, ^. EA vs. Non-acu. *P*<0.05.(C-F One-way ANOVA, followed by Tukey’s post hoc test, Data represent the mean ± SD. n = 3 rat /group)

We identified differentially expressed miRNAs in ischemic peripheral brain region samples using miRNA microarrays. qPCR was performed on selected miRNAs to validate the miRNA microarray results. Nineteen miRNAs differentially expressed on the ischemic side in both HIP CA1 and EC regions (*P*<0.05 and fold-change > 1.5) (Fig. 3A-B). The expression of Rno-miR-668 and Rno-miR-3084B-5p in the ischemic peripheral HIP CA1 and EC were both up-regulated in the EA group compared to the MCAO rats (*P*<0.05), similar to observations in the microarray expression profile (Fig. 3C, E). The expression of Rno-miR-28, Rno-miR-let-7b, Rno-miR-219a, and Rno-miR-100 decreased in both ischemic peripheral HIP CA1 and EC regions (*P*<0.05), consistent with observations in the microarray expression profile. The expression of Rno-miR-136 was up-regulated in the ischemic peripheral EC, while there was no statistical difference observed in the ischemic peripheral HIP CA1, which was not consistent with the result of the microarray expression profile compared to the MCAO rats (Fig. 3D, F).

**Fig. 4:**
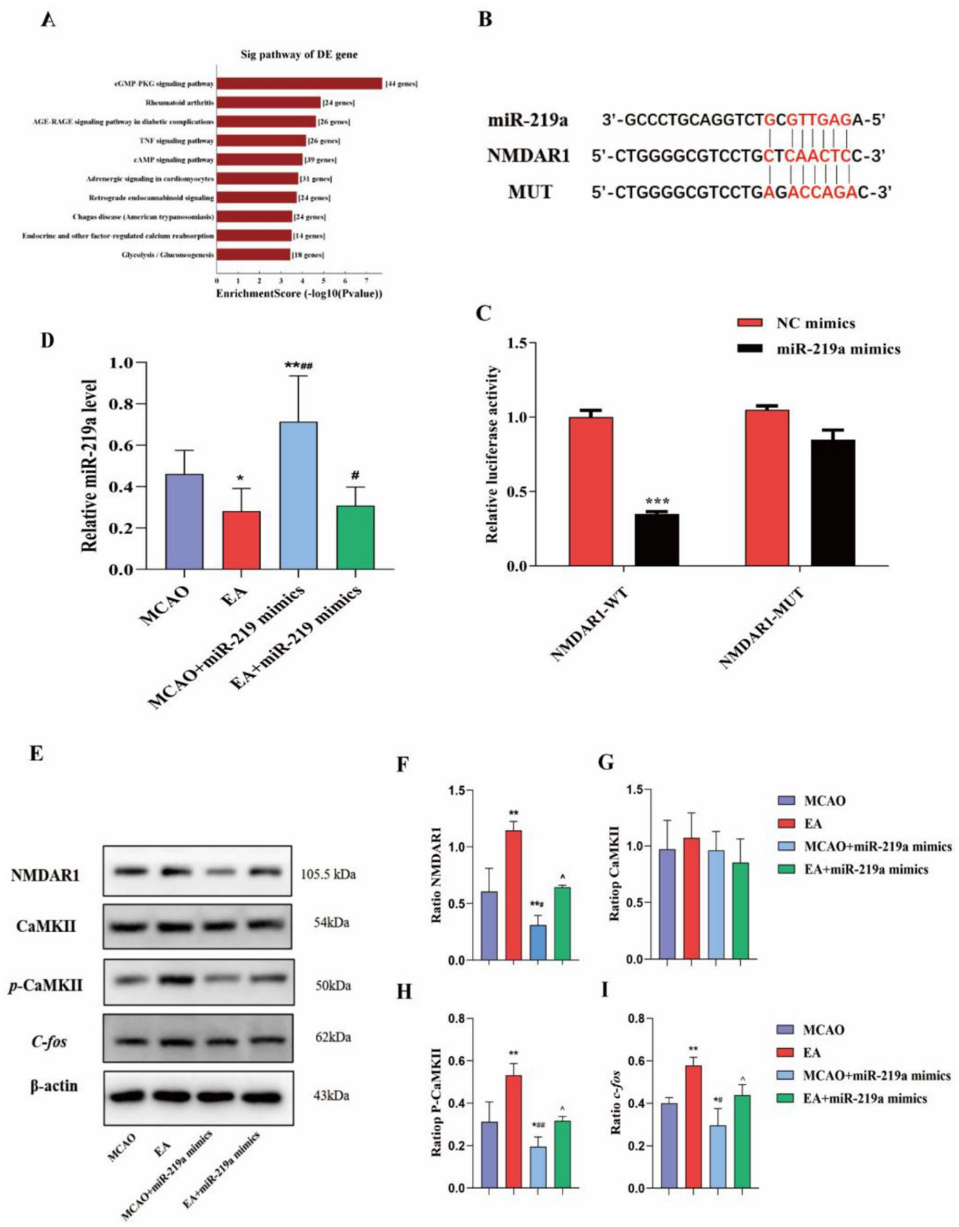
EA regulated the expression of miR-219a in the ischemic peripheral HIP and EC of MCAO rats. (**A**) Statistical analysis of GO enrichment of differentially expressed target genes and of differentially expressed miRNAs. (**B**) miR-219a-NMDAR1 interaction was detected using a dual-luciferase reporter assay. (**C**) The changes of relative luciferase activity (***, NC mimics vs miR-219a mimics, *P* < 0.01). (**D**) Relative expression of miR-219a in the HIP CA1 region. (**E**) Representative Western blot. (**F**) Western blot analysis. Significant differences in the level of NMDAR1 protein. (**G**) Significant differences in the level of retinal CaMKII protein. (**H**) Significant differences in the level of retinal *p*-CaMKII protein. (**I**) Significant differences in the level of retinal c-*fos* protein. * EA vs MCAO, MCAO+miR219a mimics VS MCAO, #. EA+miR-219a mimics vs EA, MCAO+miR-219a vs EA. *P*<0.05.(C. Independent t test., n = 3 rats/ group; D,F-I. One-way ANOVA, followed by Tukey’s post hoc test, n = 3 rats/ group, Data represent the mean ± SD)

The prediction of target genes for rno-miR-668, rno-miR-3084b-5p, rno-miR-28-5p, rno-miR-let-7b, rno-miR-7b, rno-miR-219a, rno-miR-100, and rno-miR-92a was conducted using prediction information from miRbase, miRanda, and targetscan. These tools are often used to predict the target genes of microRNAs based on complementary base pairing between the microRNA and the target gene. A total of 33,915 target genes responding to differential genes were predicted. Using the DAVID Bioinformatics Resources database (http://david.abcc.ncifcrf.gov/) for our enrichment of target genes pathway analysis, we found that the top ten signaling pathways (*P*<0.05) were: cGMP-PKG signaling pathway, rheumatoid arthritis pathway, age-rage signaling pathway, TNF signaling pathway, cAMP signaling pathway, adrenergic signaling pathway, retrograde endocannabinoid signaling, chagas disease (American trypanosomiasis), endocrine and other factor-regulated calcium reabsorption, and glycolysis/gluconeogenesis (Fig. 4A). The target gene prediction and signal transduction pathway of miRNAs that were either up-regulated or down-regulated in ischemic HIP CA1 and EC was analyzed and the cGMP-PKG (cyclic guanosine monophosphate-protein kinase G) signaling pathway (*P*<0.05) was targeted. According to the Kyoto Encyclopedia of Genes and Genomes (KEGG) data, the key nodes (NMDAR1) of the cGMP-PKG signaling pathway were found to be closely related to synaptic plasticity. Additionally, the TargetScan database predicted that miR-219a and the target gene NMDAR1 have mutually binding sites. However, it is critical to note that these predictions are based on computational analysis which requires further experimental validation to confirm their biological significance. To confirm the interaction between miR-219a and its target gene NMDAR1, 293T cell lines expressing the NMDAR1 5’UTR and NMDAR1 5’UTR mutants (Mut) were constructed using luciferase reporter vectors (Fig. 4B). The results demonstrated that NMDAR1 5’UTR protein content was significantly reduced in the presence of the miR-219a (*P*<0.01, Fig. 4C).

To validate our results in vivo, we injected miR-219a mimics into the lateral ventricle. To evaluate their efficacy, we detected the expression of miR-219a in the ischemic peripheral CA1 region of HIP in each group of rats. The results confirmed that, compared to the MCAO group, the expression of miR-219a in the EA group was reduced (*P*<0.05). When miR-219a mimics were injected into the lateral ventricle, the expression of miR-219a was further down-regulated in the ischemic area (*P*<0.05). EA further down-regulated the expression of miR-219a (Fig. 4D, P<0.05). This suggests that miR-219a may play a role in pathological processes associated with cerebral ischemia, and that EA may be a potential therapeutic strategy for modulating miR-219a expression and its associated downstream effects. The protein levels of NMDAR1 and *p*-CAMKII were reduced when miR-219a mimics were added to the lateral ventricle (*P*<0.05). After EA treatment, NMDAR1 and *p*-CaMKII protein levels were up-regulated compared to the MCAO group (*P*<0.05). CaMKII levels were unchanged across all groups (Fig. 4E-H, P>0.05). This suggests that EA may enhance endogenous NMDARs and phosphorylated CaMKII levels by regulating the expression of miR-219a.

To assess if our observations were clinically significant and to validate our findings, we measured miR-219a levels from three independent cohorts: a control group, a group of patients affected by post-stroke cognitive impairment (PSCI), and a group of patients affected by post-stroke non-cognitive impairment (PSNCI). Interestingly, miR-219a was elevated in PSCI compared to PSNCI cases (Fig. S3A).

A mini-mental state examination (MMSE) was performed alongside detection of miR-219a in each group, MMSE scores were negatively correlated with the expression of miR-219a (Fig. S3B, r^2^ = 0.2802, *P* = 0.0239).

### miR-219a regulated NMDAR-mediated synaptic plasticity in normal and ischemic brain slices

**Fig. 5:**
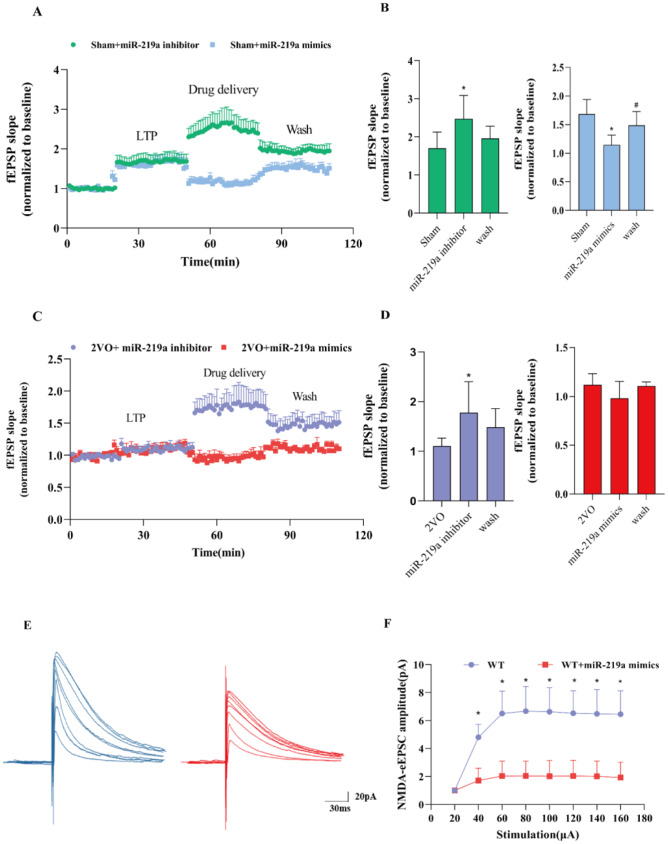
miR-219a negatively regulated NMDAR in normal and ischemic brain slices (**A**) fEPSP recording in normal-induced brain slices. Baseline recordings were taken 20 min before LTP induction, and 30 min after LTP induction. Drug delivery (green represents the miR-219a inhibitors and blue the miR-219a mimics. 30 min of induction) (**B**) fEPSP recording in 2-VO-induced brain slices. Bar graphs show the percentage change in LTP after miR-219a inhibitor treatment. *. Sham vs. miR-219a inhibitor, *P*<0.05 (left). Bar graphs show the percentage change in LTP with miR-219a mimics. *. Sham vs. miR-219a inhibitor, **#**. MCAO vs. EA, *P*<0.05 (right)C. Baseline recordings were taken 20 min before LTP induction, and 30 min after LTP induction. Drug delivery (purple represents the miR-219a inhibitors and red the miR-219a mimics. 30 min of induction). DE. Bar graphs show the percentage change in LTP after miR-219a inhibitors treatment on 2VO rats. *. 2VO vs. miR-219a inhibitor, *P*<0.05 (left) Bar graphs show the percentage change in LTP after miR-219a mimics treatment on 2VO rats (right). E. Effect of miR-219a on amplitude and area under the curve of NMDAR-mediated eEPSC. F. NMDA-eEPSC recordings in hippocampal CA1 neurons from WT and WT + miR-219a mimics *. WT vsWT + miR-219a mimics, *P*<0.05.(B, D One-way ANOVA, followed by a Tukey’s post hoc test, F. Two-way ANOVA, followed by a Tukey’s post hoc test. n = 6 sections from 3 rats/group)

In the control of our experiment, we did not add miR-219a mimics in artificial cerebrospinal fluid (ACSF). miR-219a mimics and miR-219a inhibitor were added to ACSF, respectively, in the sham and 2-VO groups to determine how miR-219a would impact long term potentiation (LTP). After a 2-hour incubation in ACSF, we recorded LTP and found that 60µM miR-219a mimics significantly inhibited LTP induced by HFS in the sham group, which was recovered after washing (Fig. 5A-D). In addition, we recorded LTP and we found that 50 µM miR-219a inhibitors significantly increased LTP in the sham group, which was recovered after washing. Identical results were observed in the 2-VO group (Fig. 5E-H). We directly added 60µM miR-219a mimics onto hippocampal slices and observed the effect of the overexpression of miR-219a on NMDAR current. NMDA-eEPSCs were measured at + 40mV holding potential in HIP CA1 neurons from the WT and WT + miR-219a mimic groups (Fig. 5I-J). We normalized the mean of the synaptic NMDAR currents evoked by the first stimulus. Unless otherwise noted, we analyzed the effects of miR-219a mimics on CA1 pyramidal cells by evoking NMDA eEPSCs at + 40 mV.

We found that overexpression of miR-219a could significantly down-regulate NMDAR current and alter its function. We examined whether the overexpression of miR-219a resulted in memory impairment. Using the Morris Water Maze Test, we evaluated spatial learning and memory ability. miR-219a mimics were injected into the lateral ventricle of the hippocampus. The Morris Water Maze Test confirmed the overexpression of miR-219a in wild type mice leading to the escape latency being significantly prolonged on days 3–4 (*P*<0.05). In addition, the number of those crossing the platform decreased significantly (*P*<0.05) and the proportion of target quadrant platform decreased significantly (Fig. S4 A-C, *P*<0.05). These findings indicate that overexpression of miR-219a impairs learning and memory-related behavior as well as LTP induction, both in vivo and ex vivo.

The Open field test (OFT) and elevated plus maze (EPM) test were used to assess anxiety-like behaviors. The results confirmed that miR-219a mimics did not experience increased anxiety in any of the groups (Fig. S4 D-G).

### MiR-219a regulated the synaptic plasticity of EC-HIP CA1 circuit to improve VCI in 2-VO rats treated with EA

**Fig. 6:**
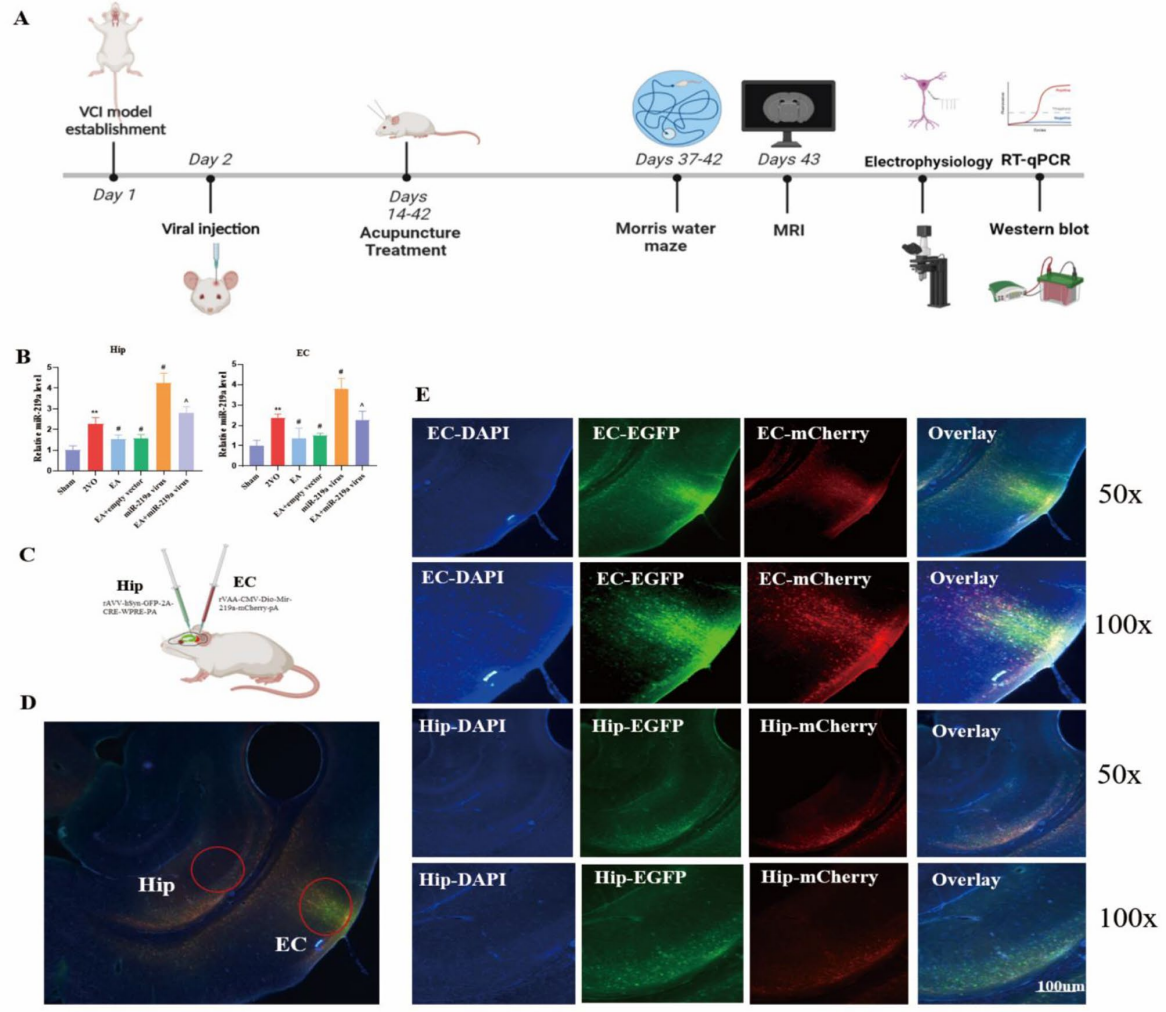
EA enhanced the synaptic plasticity of the EC-HIP CA1 circuit by miR-219a in 2-VO rats (**A**) Timeline of the experiment. (**B**) and (**C**) Relative expression of miR-219a in the CA1 region of the HIP and in the EC. (**D**) rVAA-CMV-Dio-Mir-219a-mCherry-pA injection sites in the entorhinal cortex and rAVV-hSyn-GFP-2 A-CRE-WPRE-PA injection sites in the CA1 region of the hippocampus. (**E**) Green fluorescence and red fluorescence magnified detail. *VS Sham group, *P*<0.05, **, *P*<0.01. #,VS 2-VO group, *P*<0.05. ^, VS EA group, *P*<0.05.(C. One-way ANOVA, followed by a Tukey’s post hoc test, n = 3 rats/ group, Data represent the mean ± SD)

We verified that overexpression of miR-219a leads to cognitive impairment in the MCAO model. Using the 2-VO model for validation, we assessed the results, using a timeline laid out in Fig. 6A. rVAA-CMV-Dio-Mir-219a-mCherry-pA and rAVV-hSyn-GFP-2 A-CRE-WPRE-PA retroviruses were injected in the EC and the HIP CA1 brain regions of 2-VO rats (Fig. 6C). The co-expression of the two viruses was verified under fluorescence microscopy in both the EC and the HIP CA1 region, supporting that the overexpression of miR-219a was successfully established in 2-VO rats, and confirming the presence of direct nerve fiber projection into the EC-HIP CA1 circuit (Fig. 6B). qPCR results showed that, compared to the sham group, the expression of miR-219a in the EC and HIP regions was increased in the 2-VO group (*P*<0.05), and that EA could down-regulate the expression of miR-219a (*P*<0.01). Additionally, overexpression of miR-219a could partially inhibit the effect of EA (Fig. 6D- E, *P*<0.05).

### EA regulated miR-219a to enhance synaptic plasticity in EC-HIP CA1 circuit in 2-VO rats

**Fig. 7:**
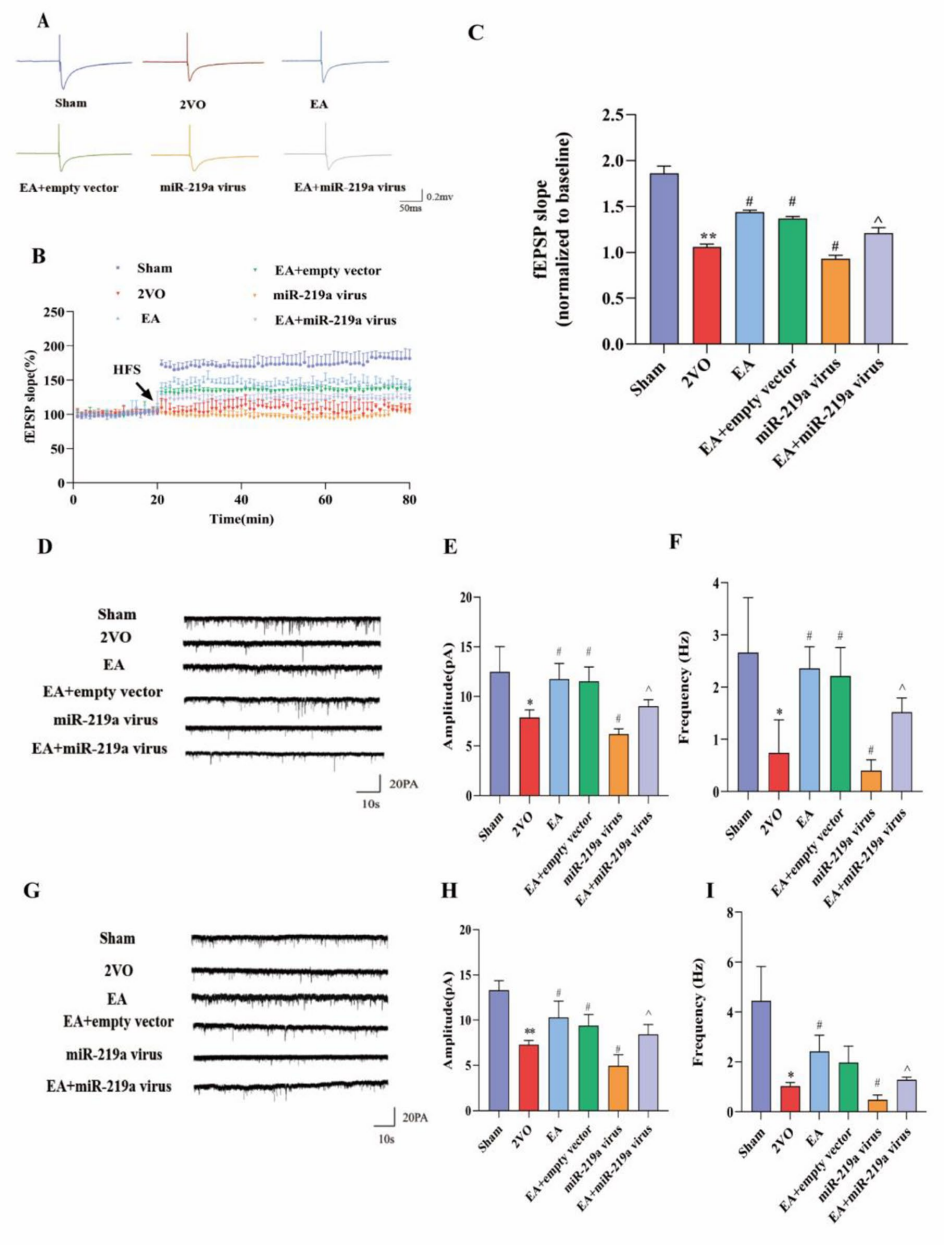
EA regulated synaptic plasticity and neuronal activity in the EC-HIP CA1 circuit (**A**) Schematic representation of electrical stimulation protocols that induce LTP at the EC-HIP CA1 circuit in 2-VO rats. (**B**) Normalized fEPSP slopes of HFS-induced LTP. (**C**) The percentage of fEPSP slope changes during 50–60 min following LTP induction in each group. (**D**) Representative sEPSC traces in the hippocampus from the experiments described. (E, F). Averaged sEPSC amplitudes and frequencies in hippocampus. G. Representative sEPSC traces from experiments described in the entorhinal cortex. (H, I) Averaged sEPSC amplitudes and frequencies in the entorhinal cortex. * VS Sham group, *P*<0.05, **, *P*<0.01. #,VS 2-VO group, *P*<0.05. ^, VS EA group, *P*<0.05. (C.E-F,H-I One-way ANOVA, followed by a Tukey’s post hoc test, n = 6 sections from 3 rats/group, Data represent the mean ± SD)

The analysis of fEPSP slopes indicated a difference among groups (n = 6 per group). The degree of LTP was represented as a percentage of the fEPSP slope (Fig. 7A). HFS of the Schaffer collateral inputs to EC-HIP CA1 pyramidal cells induced stable LTP in the slope of fEPSP in the sham group (60 min after HFS, 1.82 ± 0.09% of baseline values, Fig. 7B). In contrast, the fEPSP slope was significantly reduced in the 2-VO group (60 min after HFS, 1.07 ± 0.04 of the baseline values, *P*<0.01 vs. the sham group). However, EA could reverse the 2VO-induced LTP impairment (60 min after HFS, 1.44 ± 0.02 of baseline values, *P*<0.01 vs. the 2VO group). Compared to the EA group, the fEPSP slopes of the empty vector group were not statistically significant (60 min after HFS, 1.37 ± 0.03 of baseline values, *P*>0.05, Fig. 7C). Overexpression of miR-219a led to impaired LTP expression (60 min after HFS, 1.01 ± 0.06 of the baseline values, *P*<0.05 vs. the 2-VO group). Accordingly, EA rescue of 2VO-induced LTP impairments was eliminated by miR-219a overexpression (Fig. 7C).

sEPSC analysis determined where synaptic alterations were occurring in a circuit. Whole cell patch clamp was utilized to record excitatory postsynaptic currents of neurons in the EC and HIP CA1 regions. The results confirmed that sEPSC amplitude and frequency in the EC and HIP CA1 region were decreased in the 2-VO group (*P*<0.05). The amplitude and frequency of the EC and HIP in the EA group were higher than those in the 2-VO group (*P*<0.05). The reduced sEPSC charge reflects the weakened excitatory drive upon miR-219a overexpression (*P*<0.05). Correspondingly, overexpressed miR-219a could promote sEPSC in EA treatment (Fig. 7D-I, P<0.05). These data confirm that EA is able to enhance the excitability of EC-HIP CA1 neurons and impact synaptic plasticity by down-regulating miR-219a expression.

### EA regulated miR-219a to modulate NMDAR channel activity in 2-VO rats

**Fig. 8:**
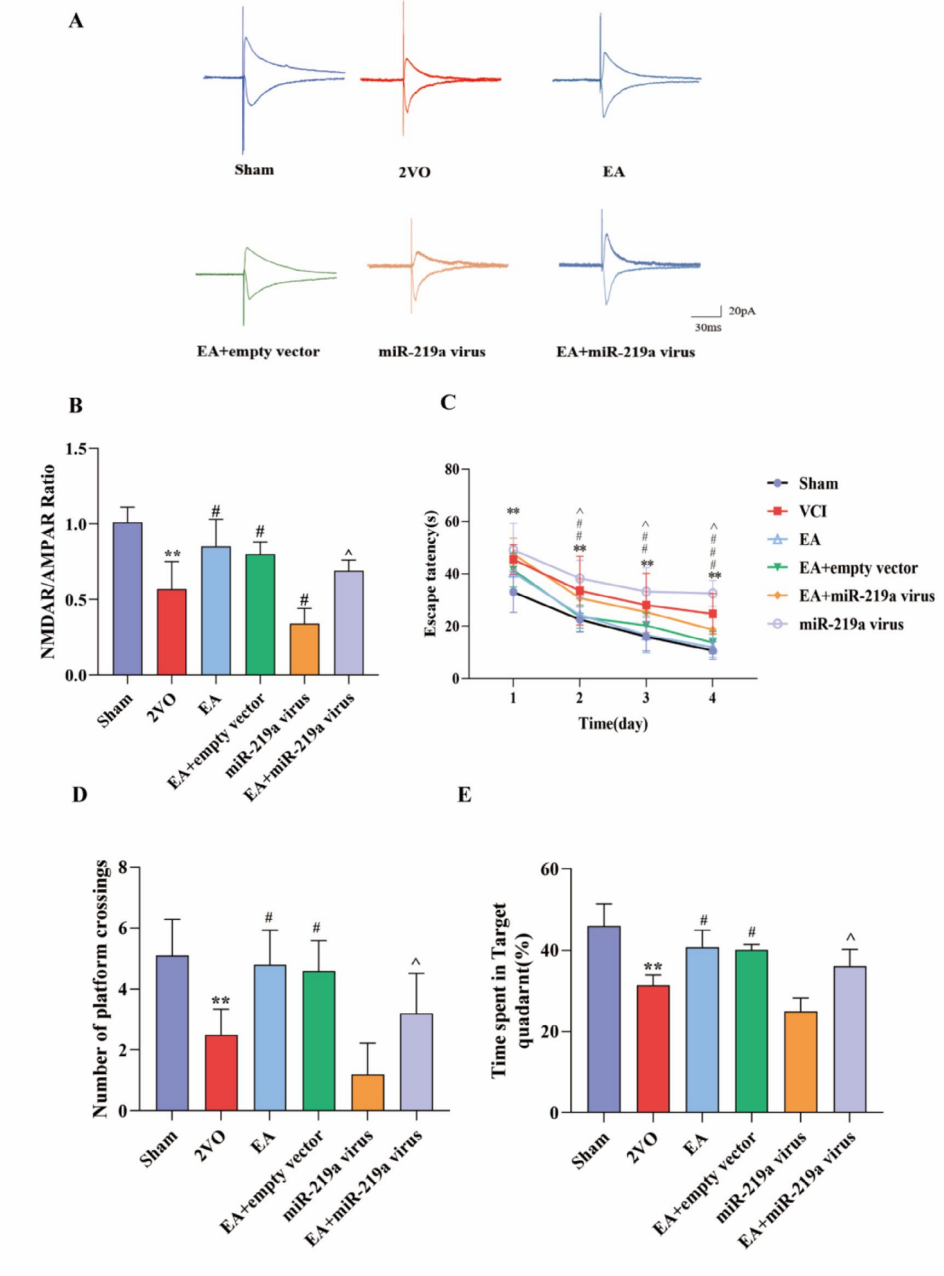
EA regulated synaptic plasticity by modulating NMDAR channel activity in the EC-HIP CA1 circuit in 2-VO rats (**A**) Representative EPSC traces in bicuculline (50 µM) at -60 mV and + 40 mV in each group. (**B**) Summary graph of the NMDAR/AMPAR ratio. (**C**) Escape latency of rats during training. (**D**) Passes over the missing platform. (**E**) Time spent in the target quadrant. *VS Sham group, *P*<0.05,**, *P*<0.01. #,VS 2-VO group, *P*<0.05. ^,VS EA group, *P*<0.05. (B. One-way ANOVA, followed by Tukey’s post hoc test, n = 6 sections from 3 rats/group, C. Repeated Measures Analysis of Variance, RM ANOVA, followed by Tukey’s post hoc test, D-E. One-way ANOVA, followed by a Tukey’s post hoc test, n = 10 rats/group, Data represent the mean ± SD)

NMDAR channel currents in the 2-VO group were significantly lower than those in the sham group (*P*<0.01). Compared to the 2-VO group, the NMDAR channel current increased in the EA group (*P*<0.05). In addition, the overexpression of miR-219a depletion reduced NMDAR-eEPSC amplitudes, EA rescued the decrease in NMDAR-eEPSC caused by the overexpression of miR-219a (Fig. 8A-B, P<0.05).

Morris Water Maze results indicated that the escape latency of the 2-VO group was significantly longer than that of the sham group (*P*<0.05). The escape latency in the EA group was reduced significantly compared to 2-VO group (*P*<0.05), while the escape latency in rats overexpressing miR-219a was prolonged further (Fig. 8C). During the test, the proportion of platform crossing times and target quadrant time in the 2-VO group was lower than in the control group (*P*<0.05). Overexpression of miR-219a decreased the swimming distance and time to platform, and increased both time in the target quadrant and crossing times (*P*<0.05). The proportion of platform crossing times and target quadrant time in the EA increased compared to the 2-VO group (*P*<0.05), and overexpression of miR-219a eliminated the effect of EA (Fig. 8D-E, P<0.05).

### 10 EA regulated miR-219a expression and activated the expression of synaptic proteins in 2-VO rats

**Fig. 9:**
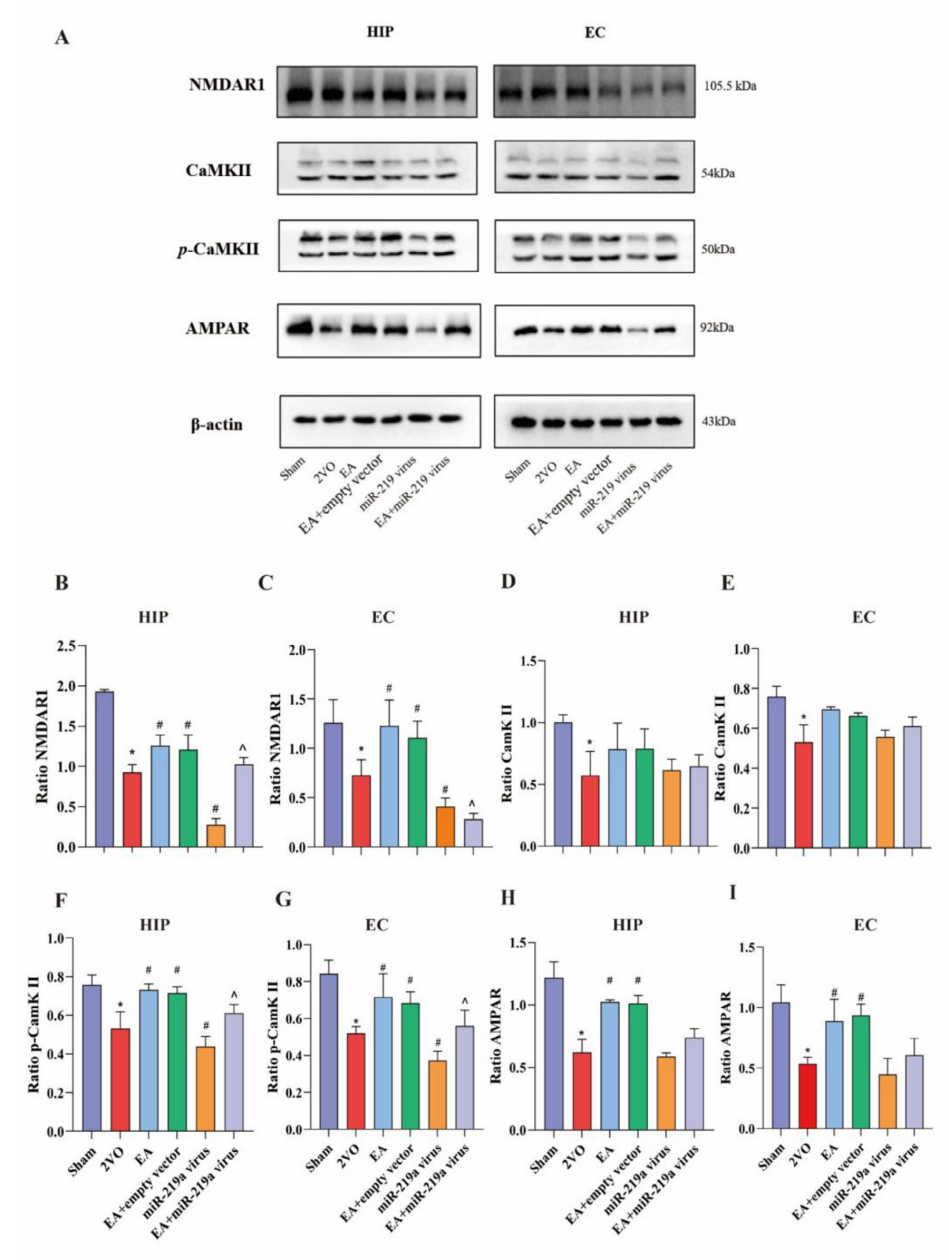
EA altered NMDAR downstream related proteins by miR-219a in 2-VO rats. **A**. Representative Western blot from each group. **B** and **C**. Western blot analysis. The level of NMDAR1 protein in the entorhinal cortex and in the hippocampus. **D** and **E**. The level of CaMKII protein in the entorhinal cortex and in the hippocampus. **F** and **G**. The level of *p*-CaMKII protein in the entorhinal cortex and in the hippocampus. **H** and **I**. The level of AMPAR protein in the entorhinal cortex and the hippocampus. *VS Sham group, *P*<0.05,**, *P*<0.01. #,VS 2-VO group, *P*<0.05. ^,VS EA group, *P*<0.05.(B-I One-way ANOVA, followed by a Tukey’s post hoc test, n = 3 rats/group Data represent the mean ± SD)

Compared to the sham group, NMDAR1,CaMKII, and AMPAR levels in the VCI group were lower. In addition, the expression of NMDAR1 and AMPAR total protein was increased in response to EA treatment compared to the 2-VO group (Fig. 9A, P<0.05), while the CaMKII level showed no statistical difference, but its phosphorylation levels were higher compared to the 2-VO group (Fig. 9F-I, P<0.05). The expression of NMDAR1 and *p*-CaMKII was downregulated in the miR-219a group, while no differences were detected in the protein expression of AMPAR (Fig. 9B-J). miR-219a overexpression negated the protective effects of EA treatment. These results indicate that miR-219a enhances synaptic plasticity in VCI through the NMDAR-dependent pathway and CaMKII phosphorylation.

## Discussion

This research focuses on clarifying the mechanism of EA improving VCI, and demonstrated that inhibition of miR-219a plays an important role in the improvement of VCI. Firstly, we observed the enhancement of ReHo activity in HIP and EC in response to EA treatment. miR-219a was confirmed to be elevated in HIP and EC in VCI, but reduced after EA treatment. In addition, NMDAR1 was identified as the target gene of miR-219a. We demonstrated that miR-219a regulates NMDAR-mediated synaptic plasticity of the EC-HIP CA1 circuit in normal and ischemic brain slices.

**Fig. 10:**
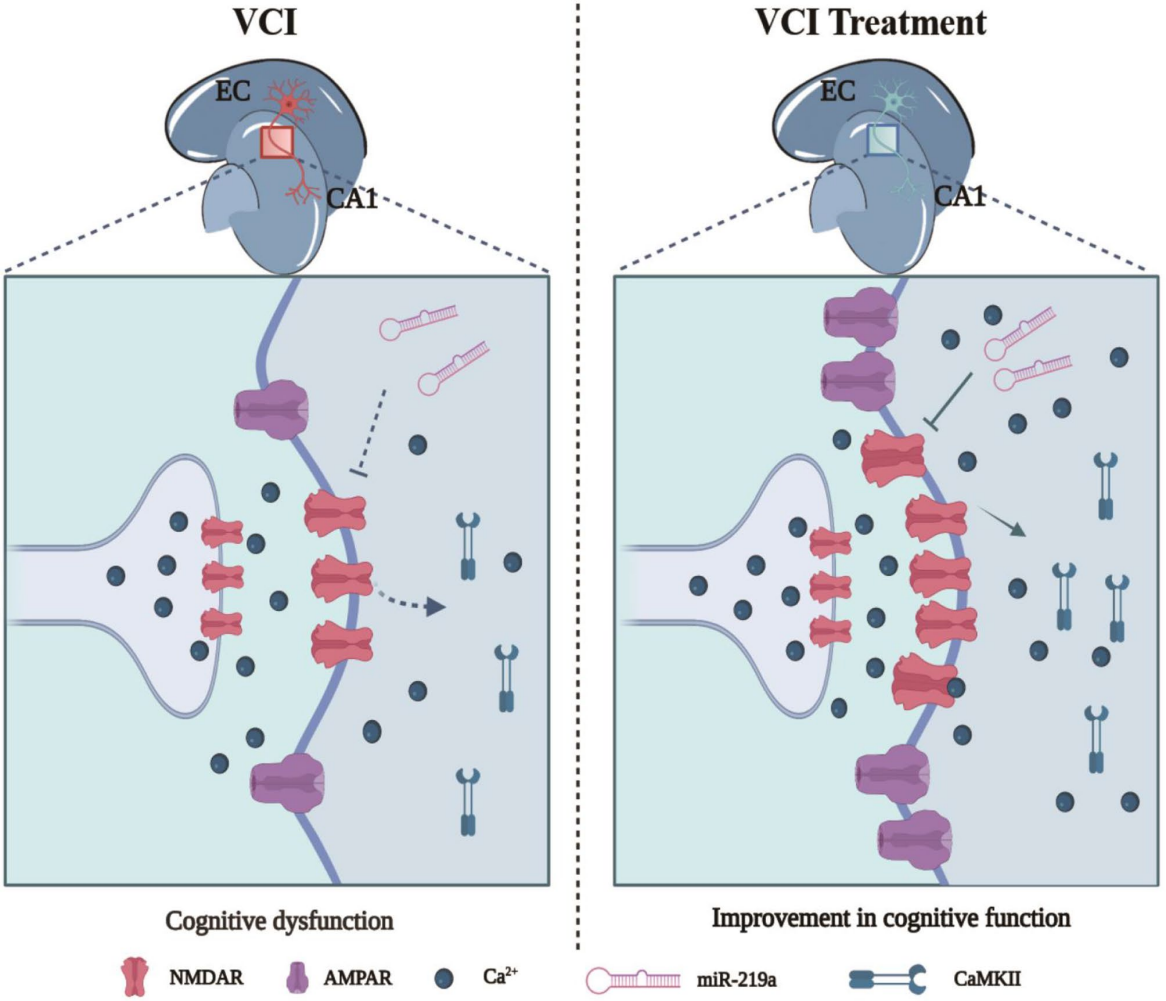
MiR-219a activation of endogenous NMDA receptor-mediated EPSC and LTP in the EC-HIP CA1 circuit improved VCI In vascular cognitive impairment, overexpression of miR-219a results in down-regulation of NMDAR1 and blocked phosphorylation of CaMKII (left). EA improves the learning and memory function of VCI by regulating miR-219a-NMDAR1 interaction, promoting CaMKII-phosphorylation, and stimulating the firing pattern of EC-HIP CA1 neurons (right)

In vascular cognitive impairment, miR-219a overexpression led to downregulation of NMDAR1 and inhibition of CaMKII phosphorylation. EA promoted CaMKII phosphorylation by regulating the interaction between miR-219a and NMDAR1, stimulated the discharge pattern of olfactory cortex-hippocampal CA1 neurons, and improved learning and memory function in VCI (Fig. 10).

### EA enhances the synaptic plasticity of EC-HIP CA1 circuit improving VCI

We developed the administration of EA at GV20 and GV24 acupoints, and found that the treatment regimen had a positive effects on VCI through clinical RCT study [17]. We found that EA could effectively improve VCI spatial learning and memory in MCAO and 2-VO animal models. In this study, rs-fMRI was used to observe that MCAO group displays reduced ReHo activity in BOLD signals in bilateral amygdala, bilateral HIP, bilateral EC, bilateral piriform cortex, bilateral sensory cortex, bilateral motor cortex, bilateral cingulate gyrus, bilateral insular cortex, bilateral dorsal thalamus, left auditory cortex, and left striatum. The local consistency of BOLD signals may reflect the strength of neuronal activity in a given brain region. The decreased level of ReHo indicates the decreased level of local neural activity, and it can be used to evaluate the damage and repair of neural activity in brain areas related to cognitive function [18].Therefore, the local consistency of the BOLD signal can be used to evaluate the level of damage and repair of neural activity in brain regions related to cognitive function. It has been reported that the volume, mean diffusion coefficient, and functional connectivity of the hippocampus are all significantly correlated with cognitive impairment in patients with post-stroke based on magnetic resonance imaging [19]. Therefore, it can be speculated that the learning and memory ability of rats with ischemic cognitive impairment and the reduction of the local activity of BOLD signal in the hippocampus are related to the impairment of spatial memory. We found in animal models that electroacupuncture can enhance the local consistency of BOLD signals in bilateral hippocampus, bilateral entorhinal cortex, bilateral amygdala, right insular cortex, right piriform cortex, and right sensory cortex in rats with vascular cognitive impairment [20]. Correspondingly, studies have shown that the amygdala is an important brain region which is responsible for emotional learning and memory, and is involved in learning, fear, and anxiety emotions and behaviors. Its function is affected by shape, and volume. The entorhinal cortex receives nerve fibers from the amygdala, and its efferent fibers project to the hippocampal region, forming the amygdala - entorhinal -hippocampal circuit . Recent studies have also suggested that the entorhinal cortex may act as a bridge between the amygdala and the hippocampus [21–22]. The entorhinal cortex may be involved in synchronous activity between ribosomes and the hippocampus. Both the amygdala and hippocampus receive connections from the entorhinal cortex, and the entorhinal stimulation induces LTP in both the amygdala and hippocampus. Transient electrical stimulation of the amygdala induces CA3-CA1 synchronization in the hippocampus in the low gamma frequency range, which is associated with improved memory [23]. This explains the increased local consistency of BOLD signals in bilateral amygdala and the improvement of spatial memory function in vascular dementia after EA treatment. The entorhinal cortex is the main medium of information exchange between the neocortex and the hippocampus [24]. There are fibrous projections between the two, constituting the entorhinal cortex-hippocampal neural circuit, which is closely related to spatial memory function [25]. Most studies suggest that synaptic dysfunction and loss lead to vascular cognitive impairment [26]. Our study illustrates that EA can enhance entorhinal cortex-hippocampal neural circuit projection, and that the improvement of synaptic plasticity in vascular cognitive impairment model rats is related to spatial learning and memory. This result is consistent with other studies, which have found that EA treatment can improve nerve cell survival and hippocampal LTP [27]. It has also been demonstrated that EA can mediate hippocampal CA1 synaptic plasticity by regulating 5-HT receptor levels [28]. Based on this, we propose that EA enhances synaptic plasticity of EC-HIP CA1 circuit and improves VCI, which assists in the treatment of cognitive dysfunction.

The targets of EA were relatively extensive, as it could activate multiple pathways and factors in the body, mobilizing various aspects of the body to play a role. The molecular network regulation characteristics of microRNAs and various intracellular biological processes had a certain fit with the multi-pathway and multi-target characteristics of EA. Our study confirmed that EA could promote the synaptic transmission efficiency of the EC-HIP neural circuit in rats after cerebral ischemia and inhibit the LTP damage. MiRNAs played a key role in synaptic plasticity. Therefore, EA was likely to improve post-ischemic stroke learning and memory impairment by mediating miRNAs to regulate the expression of target genes involved in synaptic plasticity.

### miR-219a improves VCI by regulating NMDAR in vitro and in vivo

There is documented potential of microRNAs as non-invasive diagnostic and prognostic biomarkers across a variety of pathological conditions, although many studies have identified miRNAs that play a role in the pathophysiology of brain injury [29]. However, few studies have demonstrated their function and role after acute and chronic ischemia. Our study demonstrated that EA decreased the expressions of miR-132, miR-134, miR-125b, and miR-219a in the CA1 region of the ischemic hippocampus in MCAO rats, while the expressions of miR-668 and miR-308B-5p were up-regulated in HIP CA1 and EC. Among them, miR-132 is a regulatory RNA commonly found in the cardiovascular system. In addition, miR-134 and miR-125b have been identified as dysregulated in the treatment of human cancers such as lung cancer, glioma, breast cancer, and colorectal cancer. miR-668 has been shown to inhibit mitochondrial fission-related protein MTP18, thereby inhibiting pathogenic mitochondrial fragmentation. Treatment with miR-668 mimics reduced mitochondrial fragmentation and improved renal function [30]. miR-3084B-5p was predicted to be involved in the regulation of immune-related genes [31]. Prior studies have shown that EA could regulate the abnormal expression of miR-93 and miR-124, but we did not observe these results in our study. Another study found that EA could regulate the expression of miR-93, but the focus of that study was on how EA may have impacted the MyD88/NF-κB signaling pathway by regulating miR-93 expression to enhance cognition [32]. Some studies have shown that EA can promote neural regeneration by upregulating the expression of serum miR-124. However, it should be noted that this study was conducted on MCAO model rat serum samples, while our study was conducted on the ischemic side of MCAO rat brains [33].

However, miR-219a is strongly associated with various neurodegenerative diseases [34]. Clinical studies have shown that the expression level of miR-219a in the brain tissues of 29 patients with cerebral ischemia (post-mortem) was 320 times that of the control group [35]. Additionally, in a comprehensive analysis of differential expression profiles of microRNAs in rats treated with electroacupuncture for spinal cord injury, miR-219a was also one of the five candidate biological targets [36]. NMDAR1 was further identified as a target gene of miR-219a through a dual-luciferase assay. This result is consistent with several studies which have shown that up-regulation of miR-219a can reduce LTP inhibition and neuronal apoptosis in type 2 diabetic mice by inhibiting the activation of the NMDAR signaling pathway [37].

Our results suggest that overexpression of miR-219a impairs learning and memory-related behaviors and affects entolfactory - hippocampal synaptic plasticity both in vivo and in vitro. Prior studies have found that cerebral ischemia can lead to impaired hippocampal synaptic plasticity. Maintenance of LTP requires regulation of synaptic associated protein expression. Studies have shown that response to LTP induction leads to differential expression of miRs [38]. In this study, we investigated the influence of miR-219a overexpression on the channel current changes of NMDAR using whole cell patch clamp. Previous studies confirmed that miR-219a mediated behavioral effects in mice and targeted the regulation of NMDAR signaling, consistent with our observation. Our study found that overexpression of miR-219a was negatively correlated with NMDAR, and further overexpression of miR-219a could induce impaired spatial behavior in cerebral ischemia models, confirming that up-regulation of miR-219 alters learning and memory-related behaviors in VCI models and EC-HIP synaptic plasticity by inhibiting NMDAR signaling pathways.

### NMDAR plays a dual role, with both neuroprotective and neurotoxic effects in the treatment of VCI at different times

Our study showed that miR-219 was negatively correlated with NMDAR signaling pathway-related proteins and their downstream targets. EA may enhance synaptic plasticity and spatial memory function in VCI by downregulating miR-219a expression, which activates NMDAR and downstream synaptic protein expression. However, previous research has considered NMDAR activation to be neurotoxic and harmful to synaptic plasticity. It is important to note that NMDAR is known to have a dual role as it has different therapeutic effects on VCI at different times. Reducing glutamate excitotoxicity in neurons during the acute phase has been shown to significantly reduce ischemic brain injury in rats with cerebral ischemia, while reducing glutamate-mediated excitotoxicity after cerebral ischemia has no beneficial effect on improving cerebral ischemia. In contrast, endogenous NMDAR-dependent Ca^2+^ influx triggers intracellular signal transduction cascades [39]. NMDAR is dynamically regulated through targeting and transport, and physical transport of NMDAR into and out of the synaptic membrane contributes to lasting synaptic plasticity [40]. Meanwhile, EA also had a bidirectional regulatory effect on NMDAR. Research had shown that EA treatment could promote neurogenesis and improve functional recovery. One possible mechanism for this neuroprotective effect was that EA had a regulatory effect on NMDAR [41]. Another study had also indicated that EA could activate endogenous NMDAR and induce changes in neuronal excitability [42].

Studies have demonstrated that in chronic ischemic stages, proper activation of NMDAR can promote neuron survival and support neuroplasticity, while excessive activation of NMDAR can lead to pathological results and neurodegeneration [43]. CaMKII, an abundant protein in the brain, is also an important regulator of NMDAR-mediated EPSCs and LTP [44]. CaMKII is essential for synaptic transmission and plasticity [45]. Synaptic transmission is also determined by the continued action of CaMKII. LTP was abolished in the presence of impaired CaMKII autophosphorylation [46] .We observed that CaMKII expression was not significantly different after cerebral ischemia, even after phosphorylation. Current studies indicate that the reason may be that Camkiα phosphorylation regulates NMDA receptor transport and gating, and this phosphorylation may prolong the attenuation time of NMDAR-mediated synaptic current in synapses by adjusting the sustained state of NMDAR channel opening [47]. Thus, increased CaMKII autophosphorylation is important for long-term sustained enhancement of hippocampal LTP synaptic efficacy. miR-219a may promote CaMKII by mobilizing and recruiting NMDARs at presynaptic and postsynaptic sites in HFS-induced EC-HIP LTP. Our data demonstrated that the miR-219a cascade between CaMKII and NMDARs was promoted by CaMKII phosphorylation, consistent with previous studies. We also found that AMPAR decreased after cerebral ischemia, but there was no significant difference in AMPAR expression after miR-219a overexpression. After cerebral ischemia, AMPARs in the hippocampus and cortex were also down-regulated [48]. Studies have suggested that AMPARs are related to synaptic formation and stability, and the regulation of functional AMPARs is the main mechanism of synaptic plasticity. However, recent studies have shown that LTPS in AMPAR and NMDAR can co-occur, and that NMDAR trafficking may mediate both AMPAR trafficking and AMPAR-mediated LTPS. In contrast, AMPAR trafficking does not alter NMDAR-mediated LTP. There was no significant difference in AMPAR expression after miR-219a overexpression. Therefore, we speculate that miR-219a may improve synaptic plasticity and affect cognitive function by promoting postsynaptic NMDAR expression and regulating CaMKII phosphorylation.

During HFS-induced EC-HIP LTP, miR-219a may mobilize and recruit NMDAR at both presynaptic and postsynaptic sites. These results are consistent with previous studies. We also detected that the protein expression of AMPAR and CaMKII in neurons decreases in the EC and the HIP regions. miR-219a regulates NMDAR1, altering the expression of proteins downstream of NMDAR1, and plays a role in the phosphorylation of CaMKII, while not having any role in the regulation of AMPAR expression. Increased CaMKII self-phosphorylation is important for the long-term sustained enhancement of hippocampal LTP synaptic efficacy [49]. Therefore, activation of endogenous NMDAR by miR-219a may promote synaptic plasticity of the EC-HIP CA1 circuit to improve VCI in the post-acute phase of cerebral ischemia.

We identified a relationship between miR-219a and synaptic plasticity. It is possible other that miRNAs may be involved in the process of synaptic plasticity. Our study had several limitations worth noting. Firstly, the number of clinical VCI patient samples in each group was small, and the sample size should be expanded in the future to verify the results. Second, the experimental study included a relatively small sample in each group. The novel neuroimaging and molecular tests performed could be further refined in the future.

### Conclusion

We demonstrated that EA could inhibit miR-219a, enhance synaptic plasticity in the EC-HIP CA1 circuit, increase the expression of NMDAR1, promote the phosphorylation of downstream CaMKII, and improve learning and memory abilities in rat model of VCI.

## Methods

### Experimental animal

All experimental procedures followed the International Ethical Guidelines and the National Institutes of Health Guide for the Care and Use of Laboratory Animals, and were approved by the Ethics Committee of Fujian University of Traditional Chinese Medicine [SYXK (min)2014-001 and SYXK (min)2017-001]. Male Sprague–Dawley (SD) rats (8-weeks old; weight 250–300 g) were purchased from Shanghai SLAC Laboratory Animal Co., Ltd. (Shanghai, China), weighed, and numbered after one week of adaptive feeding at the Experimental Animal Centre of Fujian University of Traditional Chinese Medicine. C57BL/6 mice used in an experiment III, which were purchased from Shanghai Slack Laboratory Animal Co. located in Shanghai, China [SYXK(Min)2019-0007].

### VCI model establishment

VCI referred to the entire spectrum of vascular brain pathologies causing cognitive deficits, ranging from subjective cognitive decline to dementia. The primary pathologies of VCI are white matter hyperintensities and infarctions. Suitable animal models of VCI can be obtained by causing Middle Cerebral Artery Occlusion (MCAO) and bilateral common carotid artery occlusion (2-VO) in rats [50].

Surgery for MCAO was performed as described previously [51]. The modified Zea Longa suture method was used to prepare the rat. 90 min after ischemia, the nylon suture was removed under anesthesia to achieve reperfusion. Control rats received sham surgery and were considered the sham operation group. The model group was neurologically scored separately 24 h after reperfusion.

2-VO surgery was performed in SD rats following methods described in previous studies [52]. The 2-VO rat model was obtained performing permanent occlusion of the bilateral common carotid artery, while the rats were anesthetized intraperitoneally with 40 mg/kg of sodium pentobarbital. During anesthesia, the neck skin of rats from both groups was cut longitudinally using surgical scissors, one side of the common carotid artery was separated. The common carotid artery was then ligated using a nylon cord. After five minutes, the other common carotid artery was separated and ligated in the same manner. In the sham operation group, the operation was performed identically, with the exception of arterial ligation. The BCAS surgery was performed with minor modifications in accordance with the literature. In short, the mice were anesthetized with 2% isoflurane delivered in medical oxygen through a mask. Both common carotid arteries were then exposed and isolated from the vagus nerve. Microcoils with an internal diameter of 0.18 mm (Anrui Biotechnology, Xi’an, China) were used in the surgery. As described elsewhere, a 0.18 mm microcoil was wound around the right common carotid artery. After one hour, a 0.18 mm microcoil was wound around the left common carotid artery. The sham-treated animals were exposed to the same procedure, except that the microcoils were not placed around the arteries [53].

### Experimental design and animal grouping

In experiment I, male SD rats were randomly placed into a model group and a sham-operated group (sham group), the model group was divided into the MCAO, EA, and non-acupoint (non-acu) groups (n = 18/group). Morris Water Maze Tests were used to assess memory and learning abilities. MRI was used to detect changes in the BOLD signal ReHo in MCAO rats. LTP was used to detect synaptic plasticity in the entorhinal cortical-hippocampal CA1 circuit. miRNA chips were used to select for differentially expressed miRNAs in MCAO rats, which were then validated using quantitative real-time PCR (qPCR). We used the results of experiment I to explore if EA improved cognitive dysfunction in learning and memory in MCAO rats.

In experiment II, the MCAO rat model was established as in experiment I. The MCAO rats were randomly divided into 4 groups: MCAO group, EA group, MCAO + miR-219a mimic group, and EA + miR-219a mimic group (n = 8/group). An injection was performed through the ischemic ventricle under the guidance of the stereoscopic brain locator in rats, and the administration time was 30 min before modeling. Dual luciferase verified the interaction between miR-219a and NMDAR1. Correspondingly, we detected the expression level of miR-219a in the CA1 region of the hippocampus on the ischemic side of rats in each group. Western blotting indicated the expression of learning- and memory-related proteins. Experiment II confirmed that electroacupuncture regulation of miR-219a improved the expression of learning- and memory-related proteins in MCAO rats.

In experiment III, the mice were randomly assigned to two groups: sham and 2-VO. Brain slices obtained from rats in both groups were added to miR-219a mimics and miR-219a inhibitors in electrophysiological experiments and divided into four groups: sham + miR-219a mimics group, sham + miR-219a inhibitor group, 2-VO + miR-219a mimics group, and 2-VO + miR-219a inhibitor group (n = 6/group from three mice for each group). The findings of this experiment indicate that miR-219a mimics LTP induction.

To measure NMDAR-mediated currents, miR-219a mimics were added to the lateral ventricle in wild-type mice, (n = 6 sections from three mice for each group). The Morris Water Maze Test was used to assess the learning and memory function (n = 5). We used the results of experiment III to determine if miR-219a could regulate NMDAR-mediated synaptic plasticity in normal and ischemic brain slices.

In experiment IV, to explore how EA mediated miR-219a may regulate NMDAR in the EC-HIP circuit, 2-VO rats were randomly assigned to six groups (n = 10/group): (I) 2-VO group, (II) 2-VO + EA group (EA group), (III) 2-VO rats injected with rVAA-CMV-Dio-Mir-219a-mCherry-pA and rAVV-hSyn-eGFP-2 A-CRE-WPRE-PA (miR-219a virus group), (IV) 2-VO rats that received EA treatment injected with rVAA-CMV-Dio-Mir-219a-mCherry-pA and rAVV-hSyn-eGFP-2 A-CRE-WPRE-PA (EA + miR-219a virus group), and (V) 2-VO rats that received EA treatment injected with control virus and hSyn-eGFP-2 A-CRE-WPRE-PA (EA + empty vector group). The Morris Water Maze Test was used to assess the learning and memory function. LTP was used to detect synaptic plasticity in the entorhinal cortical-hippocampal CA1 circuit in 2-VO rats. Detection of EC-HIP pyramidal neuron spontaneous excitatory postsynaptic currents (sEPSC) events was performed and whole-cell NMDAR channel currents were recorded. We subsequently measured NMDAR and CaMKII expression, using the results of experiment III to explore the underlying mechanism of EA stimulation and miR-219a-NMDAR in improving the cognitive function of VCI.

### Virus injections

In experiment II, miR-219a mimics were diluted to 10 mM and stored at -20℃ before injection following protocols presented in previous studies [54]. The method of lateral ventricle localization and intraventricular injection in rats was referred to relevant literature. The administration time was 30 min before the modeling [55].In experiment III, In the vitro experiment, we did add miR-219a mimics and miR-219a inhibitors in ACSF. This study adopted the kit of Shanghai genepharma Company (http://www.genepharma.com/). The recommended dosage is 30–100µmol/L. Therefore, we determined the optimal concentration of miR-219a through previous literature search and pre-experiment [56, 57]. We have selected a concentration of 60µmol/L for miR-219a mimics and 50µmol/L for miR-219a inhibitors. In vivo experiment, the method of lateral ventricle localization and intraventricular injection in mice was referred to relevant literature [58].

In experiment IIII, the viral tools were prepared by Brain VTA (Brain VTA Co, Ltd., Wuhan, China), and viruses were injected into the brains of rats. Rats in the EA + empty vector group were injected with rVAA-CMV-Dio-mCherry-pA (serotype 1, 1 × 10^13^ particles/mL) in the Entorhinal cortex (from Bregma AP:-6.50 mm, ML: -7.00 mm, DV: -6.38 mm) and rAVV-hSyn-eGFP-2 A-CRE-WPRE-PA (retrotype, 2 × 10^12^ particles/mL) in the hippocampal CA1 region (from Bregma AP:-6.00 mm, ML:- 5.50 mm, DV: −6.38 mm), while rats in the miR-219a virus group and EA + miR-219a virus group were injected with rVAA-CMV-Dio-Mir-219a-mCherry-pA (serotype 1, 1 × 10^13^ particles/mL) in the Entorhinal cortex (from Bregma AP:-6.50 mm, ML: -7.00 mm, DV: -6.38 mm) and rAVV-hSyn-GFP-2 A-CRE-WPRE-PA (retrotype, 2 × 10^12^ particles/mL) in the hippocampal CA1 region (from Bregma AP:-6.00 mm, ML:-5.50 mm, DV: -6.38 mm). The virus was delivered using a syringe pump at a rate of 40 nL/minute for ten minutes, for a total of 400 nL/infusion. The syringe was then raised 2 mm, and left in place for 15 min after each injection to allow for virus diffusion, and was then slowly retracted.

Rats were deeply anesthetized with sodium pentobarbital and perfused intracardially with saline, followed by a fixative (Paraformaldehyde 4%) (PFA, 16,005, Sigma-Aldrich). Brains were removed and post-fixed overnight for 24 h. Free-floating Sect. (40 μm thick, one-fourth of the total sections that contained the hippocampus-CA1 and EC) were obtained to observe the Entorhinal cortex - hippocampal CA1 using a laser scanning confocal microscope (LSM 710, Carl Zeiss, Germany).

### Neurological scoring

A modified neurological severity score (mNSS) was used, and is a composite of motor, sensory (visual, tactile, and proprioceptive), reflex, and balance tests. Function was graded on a scale of 0 to 14 (normal = 0; maximal deficit = 14), where 1 point is awarded for each inability to perform a task.

### EA Treatment

EA stimulation was applied at the Baihui (G20) acupoint (on the middle of the parietal bone, 2 mm obliquely backward) and the Shenting (G24) acupoint (on the anterior midline, in front of the frontal-parietal suture junction, 2 mm obliquely backward) using a 0.5 mm needle (No. 30 Hua Tuo Brand), once a day for 30 min, five days/week. The MCAO group of experiment I received EA treatment for two weeks, the 2-VO group of experiment II received EA treatment for four weeks.

An EA therapeutic apparatus (G6805, Hua Tuo Brand) was used, and the stimulation parameters were set to 6 V, 1–3 mA, sparse-dense waves, and 2/20 Hz (fig S1A).

The non-acu group used was subjected to the same stimulation with the same parameters at non-acupoints (on the lower part of the bilateral flank, 3 mm obliquely downward). The sham and model groups were kept under the same conditions and then returned to their cages for rearing [59].

### Behavioral assessment of learning and memory

#### Step-down test

In the step-down test, mice were placed on a platform and if the mice stepped down onto the floor they received a 36 V AC foot shock. Mice typically jumped quickly onto the platform to avoid the electric stimulation. The error number (more than two extremities touching the grid) and the electric shock time were recorded. One day later, the mice were placed on the platform again without electrifying the grid. The step-down latency and the time remaining on the platform were recorded over a five-minute period [60].

#### Morris water maze

The Morris Water Maze test (Chinese Academy of Sciences, Beijing, China) was used to assess cognitive ability. The maze consisted of a 100 cm diameter circular pool filled with water at 25℃ and a 7 cm diameter escape platform placed in the center of the designated target quadrant, approximately 5 mm below the water level. The rats were trained allowing them 90 s to find the platform four times per day for five days. The distance swam, number of crossings, position of the target platform as well as the other three platforms, and time spent in the quadrants of the four platforms were measured [60].

### Observation and volume measurement of cerebral infarction

#### Rat MRI detection

We used MRI to detect the percentage of rat cerebral infarction volume in the cerebral hemispheres. MRI was performed at Fujian University of Traditional Chinese Medicine, using a Bruker Biospec 7.0T (70/20USR MRS scanner, Bruker Biospin, Germany). Before MRI scanning, the rats were subjected to isoflurane anesthesia, and were fixed on the magnetic resonance head frame. The anesthetic gas was continuously flowed through the nasal cannula to keep the body temperature of the rats at 37 °C. T2-weighted turbo positive pressure scan was performed using small animal MRI.

The acquisition parameters were as follows: T2-weighted images (T2WI) using TurboRARE sequence (TR/TE = 4,200/35 ms, field of view = 20 × 20 mm, averages = 4, image size = 256 × 256, slices = 30, slice thickness = 0.5 mm), rs-fMRI using an echo-planar imaging (EPI) sequence (TR/TE = 2,000/10.28 ms, field of view = 20 × 20 mm, repetitions = 200, image size = 64 × 64, slices = 30, slice thickness = 0.5 mm). Images were collected, and Image J image processing software was employed to calculate the volume and percentage of cerebral hemispheres of cerebral infarction [61].

#### MRI scanning and analysis

MRI used the paramagnetic properties of deoxyhemoglobin. The paramagnetic properties of deoxyhemoglobin were utilized to measure the outcome value R2* (apparent relaxation rate, expressed per second), with higher values corresponding to higher local deoxyhemoglobin levels and lower oxygenation. Fifteen T2-weighted MR images were acquired at one coronal slice using a combination sequence for Bold-MRI analysis, which has been previously described. The MRI was performed on a 3-T whole-body magnetic resonance system (Magnetom Prisma, Siemens Medical Systems, Erlangen, Germany) [62]. The images were then imported for further analysis using MATLAB version 7.11. The original Bruker images were also converted to DICOM format using Paravision for further analysis.

#### Regional homogeneity (ReHo) analysis

ReHo is used to measure the local temporal similarity between a particular voxel and its neighbors, and to assess the strength of functional synchronization in local brain regions. We used Kendall’s coefficient of concordance (KCC) to define the ReHo value of the center voxel. Individual ReHo maps were generated by calculating the KCC value of the time series for a given voxel with its nearest neighbors, and smoothed for further statistical analysis by Gaussian Kernel with a 1.5× voxel size Full Width of Half Maximum (FWHM) to improve the signal-to-noise ratio (SNR). Voxel-wise statistics were performed using one-way analysis of variance (ANOVA) to determine the difference across all groups [63].

### Electrophysiology

Candidate MSNs in the dorsolateral striatum were identified using infrared differential interference contrast video microscopy (Eclipse FN1; NIKON Japan). Patch pipettes (3–5 MΩ) were constructed from borosilicate glass capillaries pulled on a P-1000 micropipette puller (Sutter Instruments, Novato, CA, USA). Whole-cell patch clamp recordings were conducted in gap-free acquisition mode with a sampling rate of 10 kHz and low-pass filtered at 3 kHz, using a Multi-Clamp 700B amplifier, Digidata 1550 digitizer, and *p*Clamp 10.6 software (Molecular Devices, Sunnyvale, CA, USA). The access resistance was monitored continuously during the experiments. Cells were excluded if their access resistance was>25 MΩ.

Artificial cerebrospinal fluid (ACSF, 1000 mL) containing 126mM NaCl, 2.5mM KCl, 125mM NaH_2_PO_4_, 26mM NaHCO_3_, 10mM D-glucose, 0.5mM CaCl_2_, and 10mM MgSO_4_ was prepared before the experiment, and 300 mL was frozen for 60 min. The experimental animals were anesthetized using 5% sodium pentobarbital and immediately decapitated. The rat brain tissue was placed on the plane of the slice slot holder with glue, and immersed in a tank filled with an ACSF ice-water mixture and oxygen (95% O_2_ + 5% CO_2_ mixture). The brain tissue was cut into 300–400 μm slices using a Leica vibrating microtome and transferred to an isolated brain slice incubator. After incubating at a constant temperature in a water bath at 31℃ for 30 min, the patch clamp field potential was recorded. When the recording electrode was put in contact with the ACSF, the system was adjusted to the current clamp mode (I = 0), the liquid junction potential was adjusted to near 0, and the resistance of the recording electrode was checked. The resistance of the recording electrode was required to be 3–6 Ω. The stimulation electrode was set at 1–7 µA, with a 20-second interval stimulation square wave, and the field excitatory postsynaptic potential (fEPSP) was recorded. In the LTP test, 30% electrical stimulation of the maximum peak value was used as the baseline field potential stimulation intensity (stimulation was given once in 20 s, and the baseline was recorded for 20 min). After baseline recording, 100 Hz high-frequency stimulation was applied through the stimulation electrode twice to induce LTP (with an interval of 30 s between the stimulations), basic field potential stimulation was continued, and fEPSP was recorded for 60 min. For spontaneous excitatory postsynaptic current (sEPSC) recording, samples were voltage-clamped at -70 mV in the presence of picrotoxin (50 µM). The internal electrode fluid for resting membrane potential and sEPSC recording contained 140mM K-gluconate, 3mM KCl, 2mM MgCl_2_, 0.2mM EDTA, 10mM HEPES, and 2mM ATP (Na^+^ salt), with pH adjusted to 7.2 and osmotic pressure adjusted to 280–290 mOsmol/L. In the whole-cell recording mode, the voltage was held at -70mv, and the sEPSC of pyramidal neurons was recorded. The total recording time for each cell was three minutes, and data from the central minute (60 to 120 s) were used for analysis [64].

### Fluorescence labeling and chip hybridization for RNA

Total RNA was extracted from ischemic hippocampus and entorhinal cortex tissues of the model and electroacupuncture groups. After RNA quality detection, total RNA of the model group was pseudo-labeled with the specific fluorescent label Cy3. The total RNA of the electroacupuncture group was labeled with Cy5, and 8 chips were prepared.

We performed hybridization image acquisition and data analysis using a biochip laser scanner. The parameters of the biochip laser scanner were adjusted according to the signal intensity of exogenous quality control and endogenous quality control. Split-tiff software was used to split the composite images of the two fluorescence images into monochromatic fluorescence images. The images were imported into the array-Pro gene chip analysis software. After both automatic and manual positioning and arrangement, the range of hybridization points could be found, background noise was filtered, fluorescence signal intensity values of gene expression were extracted and output in the form of a list, and the scanned images were converted into values. The output data parameters included signal strength, signal-to-noise ratio, and background. Data standardization processing was undertaken because of the imbalance of sample difference, detection rate and fluorescence labeling efficiency, the original extraction signals of Cy5 and Cy3 needed to be equalized and corrected [65].

### Dual-luciferase reporter vector

(1) Grin1 5 ‘UTR sequence: 5’ -CTGGGGCGTCCTGCTCAACTCC-3’ and Grin1 mutant 5’ UTR: 5 ‘-CTGGGGCGTCCTGAGACCAG-3’ were both cloned individually;

(2) Grin1 target fragment was linked to pSI-CHECK2 vector to construct PSI-Check2-GRIN1 3’URT and PSI-Check2-PRKE2 3’URT_MUT, which was verified by sequencing;

(3) 293T cells were co-transfected with miR-219a mimics and the newly constructed vector;

(4) The Luciferase Reporter Assay System (E1910) was used to detect the Luciferase activity of different groups of cells after lysis [66].

### Western blot

Western blotting was performed using standard methods. Protein concentration was assayed after extraction of bilateral hippocampal tissue protein supernatant. The concentration of the extracted protein was determined using the bicinchoninic acid (BCA) protein detection kit (23,227, Thermo Fisher Scientific, USA). The steps included protein extraction, determination of protein concentration using a BCA kit, electrophoresis, membrane washing and blocking, incubation with primary and secondary antibodies, visualization of immunoreactive bands, and densitometric analysis using imaging software [67].

### Real-time quantitative PCR (qPCR)

The miRNA All-in-One one™ qRT-PCR miRNA Detection kit was used for reverse transcription reaction and PCR amplification. The reactions were performed using an ABI 7500 fluorescence quantitative PCR machine following the manufacturer’s instructions. The following thermocycling conditions were used for PCR: initial denaturation at 95˚C for 5 min; 40 cycles of 30 s at 95˚C, 45 s at 56.5˚C, and 30 s at 72˚C; final extension at 72˚C for 5 min. The expression levels of target genes were calculated by relative quantification using the 2^−∆∆CT^ method [68].

### Statistical analysis

Experimental data were processed using SPSS 20.1 statistical software, and all measurement data were expressed as mean ± standard deviation. Analysis of variance was used to compare different groups, and LSD was used for further comparison. Comparison between the two groups was performed using a two independent sample T-test.

## Electronic supplementary material

Below is the link to the electronic supplementary material.

**Figure S1**. EA could improve the cognitive function of MCAO rats A. Schematic representation of electroacupuncture points. B. Timeline and stages of the intervention design. C. Neurological deficit scores before treatment. D. Neurological deficit scores 24 hours after treatment. E. Neurological deficit scores 7 days after treatment. F. Neurological deficit scores 14 days after treatment. G. Results from the step-down avoidance test for each group before treatment. H. Results from the step-down avoidance test for each group 14 days after treatment. I. Escape latency in the Morris water maze test in each group. J. Morris water maze - visible platform task. K. Percentage of time spent in the target quadrant. *VS Sham group, P<0.05,**, P<0.01. #,VS 2-VO group, P<0.05. ^,VS EA group, P<0.05.(C-H, J-K,One-way ANOVA, followed by Tukey?s post hoc test, Data represent the mean ± SD. n= 18 rat /group, I . C. Repeated Measures Analysis of Variance, RM ANOVA, followed by Tukey?s post hoc test, n=10 rats/group, Data represent the mean ± SD). **Figure S2**. Effect of EA on cerebral infarction in MCAO rats. A. Size quantification of the infarcted brain region in MCAO group. B. Size quantification of the infarcted brain region in EA group. C. Size quantification of the infarcted brain region in Non-acu group. D. Determination of the infarct area in each group. (the dotted line shows the boundary of hippocampus brain regions) *. Sham vs MCAO, #.MCAO vs EA , ^.EA vs Non-acu. P<0.05.( D. One-way ANOVA, followed by Tukey?s post hoc test, n= 18 rat /group, Data represent the mean ± SD). **Figure S3** The cognitive function of PSCI is negatively correlated with the expression of miR-219a. A. Identification of serum miRNA signature change in PSCI and PSCIND group. B. Correlation analysis was conducted between the levels of miR-219a and MMSE score was identified. *, VS PSCIND group , P<0.05,**, P<0.01.(A. Independent t test., PSNCI group n=10, PSCI group=9; B linear correlation analysis?Data represent the mean ± SD?PSNCI group n=10, PSCI group=9). **Figure S4**. miR-219a negatively regulates NMDAR and affects learning and memory A. Escape latency in the Morris water maze test in WT and WT+miR-219a group. B. Morris water maze - visible platform task. C. The percentage of time spent in the target quadrant. D-E. Anxiety levels were measured using the elevated plus maze. F-G. OFT refers to Open Field Test. Time in the center measures anxiety levels. WT: wild type NS: non-significant.(A. Repeated Measures Analysis of Variance, RM ANOVA, B-G. Independent t test. n = 5 rats/ group, followed by Tukey?s post hoc test,). **figure S5** flowchart of the experimental design

## Data Availability

The datasets used and/or analyzed during the current study are available from the corresponding author upon reasonable request.
