# Supplementary material for: Electroacupuncture protective effects after cerebral ischemia are mediated through miR-219a inhibition
Source: Biol Res. 2023 Jun 30;56:36. doi: 10.1186/s40659-023-00448-z (PMC10311837; doi:10.1186/s40659-023-00448-z)
Supplement: Supplementary file 1 — Figure S1. EA could improve the cognitive function of MCAO rats A. Schematic representation of electroacupuncture points. B. Timeline and stages of the intervention design. C. Neurological deficit scores before treatment. D. Neurological deficit scores 24 hours after treatment. E. Neurological deficit scores 7 days after treatment. F. Neurological deficit scores 14 days after treatment. G. Results from the step-down avoidance test for each group before treatment. H. Results from the step-down avoidance test for each group 14 days after treatment. I. Escape latency in the Morris water maze test in each group. J. Morris water maze - visible platform task. K. Percentage of time spent in the target quadrant. *VS Sham group, P<0.05,**, P<0.01. #,VS 2-VO group, P<0.05. ^,VS EA group, P<0.05.(C-H, J-K,One-way ANOVA, followed by Tukey?s post hoc test, Data represent the mean ± SD. n= 18 rat /group, I . C. Repeated Measures Analysis of Variance, RM ANOVA, followed by Tukey?s post hoc test, n=10 rats/group, Data represent the mean ± SD). Figure S2. Effect of EA on cerebral infarction in MCAO rats. A. Size quantification of the infarcted brain region in MCAO group. B. Size quantification of the infarcted brain region in EA group. C. Size quantification of the infarcted brain region in Non-acu group. D. Determination of the infarct area in each group. (the dotted line shows the boundary of hippocampus brain regions) *. Sham vs MCAO, #.MCAO vs EA , ^.EA vs Non-acu. P<0.05.( D. One-way ANOVA, followed by Tukey?s post hoc test, n= 18 rat /group, Data represent the mean ± SD). Figure S3 The cognitive function of PSCI is negatively correlated with the expression of miR-219a. A. Identification of serum miRNA signature change in PSCI and PSCIND group. B. Correlation analysis was conducted between the levels of miR-219a and MMSE score was identified. *, VS PSCIND group , P<0.05,**, P<0.01.(A. Independent t test., PSNCI group n=10, PSCI group=9; B linear correlation analysis?Data repres [file 40659_2023_448_MOESM1_ESM.docx]

**Figure S1. EA could improve the cognitive function of MCAO rats**

A. Schematic representation of electroacupuncture points. B. Timeline and stages of the intervention design. C. Neurological deficit scores before treatment. D. Neurological deficit scores 24 hours after treatment. E. Neurological deficit scores 7 days after treatment. F. Neurological deficit scores 14 days after treatment. G. Results from the step-down avoidance test for each group before treatment. H. Results from the step-down avoidance test for each group 14 days after treatment. I. Escape latency in the Morris water maze test in each group. J. Morris water maze - visible platform task. K. Percentage of time spent in the target quadrant.*VS Sham group, *P*＜0.05,**, *P*＜0.01. #,VS 2-VO group, *P*＜0.05. ^,VS EA group, *P*＜0.05.(C-H, J-K,One-way ANOVA, followed by Tukey’s post hoc test, Data represent the mean ± SD. n= 18 rat /group, I . C. Repeated Measures Analysis of Variance, RM ANOVA, followed by Tukey’s post hoc test, n=10 rats/group, Data represent the mean ± SD)

**Figure S2. Effect of EA on cerebral infarction in MCAO rats.**

A. Size quantification of the infarcted brain region in MCAO group. B. Size quantification of the infarcted brain region in EA group. C. Size quantification of the infarcted brain region in Non-acu group. D. Determination of the infarct area in each group. (the dotted line shows the boundary of hippocampus brain regions) *. Sham vs MCAO, #.MCAO vs EA , ^.EA vs Non-acu. *P*＜0.05.( D. One-way ANOVA, followed by Tukey’s post hoc test, n= 18 rat /group, Data represent the mean ± SD)

**Figure S3 The cognitive function of PSCI is negatively correlated with the expression of miR-219a.**

A. Identification of serum miRNA signature change in PSCI and PSCIND group. B. Correlation analysis was conducted between the levels of miR-219a and MMSE score was identified. *, VS PSCIND group , *P*＜0.05,**, *P*＜0.01.(A. Independent t test., PSNCI group n=10, PSCI group=9; B linear correlation analysis，Data represent the mean ± SD，PSNCI group n=10, PSCI group=9)

Table S2 :

| Table 1. Baseline Characteristics of Patients | | | | |
| --- | --- | --- | --- | --- |
| Variable | PSCI Group  (n =9) | PSNCI Group  (n =10) | *t/Z* value | *P* value |
| Demographic Information | | | | |
| Age, mean (SD), y | 61.00 (4.06) | 59.4 (4.77) | 0.782 | 0.444 |
| Female sex, No. (%) | 3 (33.33%) | 3 (30.00%) | 0.106 | 0.783 |
| Body mass index, mean (SD) | 24.23 (5.11) | 23.15 (2.75) | -0.435 | 0.567 |
| Education, mean (SD), y | 6.56 (3.45) | 6.74 (3.67) | -0.123 | 0.881 |
| Symptom duration, mean (SD),d | 38.61 (40.35) | 29.99 (49.56) | -0.159 | 0.829 |
| Type of Ischemic stroke, No. (%) | 9 (100%) | 10 (100%) | - | - |
| Scale | | | | |
| MMSE, mean (SD),score | 16.10 (4.60) | 27.33 (1.58) | 6.941 | <0.001 |

**Figure S4. miR-219a negatively regulates NMDAR and affects learning and memory**

A. Escape latency in the Morris water maze test in WT and WT+miR-219a group. B. Morris water maze - visible platform task. C. The percentage of time spent in the target quadrant. D-E. Anxiety levels were measured using the elevated plus maze. F-G. OFT refers to Open Field Test. Time in the center measures anxiety levels. WT: wild type NS: non-significant.(A. Repeated Measures Analysis of Variance, RM ANOVA, B-G. Independent t test. n = 5 rats/ group, followed by Tukey’s post hoc test,)

**Table S2.**

| Brain areas | MCAO  VS Sham | | EA  VS MCAO | | Non-acu  VS MCAO | | EA  VS Non-acu | |
| --- | --- | --- | --- | --- | --- | --- | --- | --- |
|  | Clusters | T value | Clusters | T value | Clusters | T value | Clusters | T value |
| Amygdala nucleus（left） | 117 | -5.6582 | 25 | 3.746 | 17 | 3.5511 | - | - |
| Amygdala nucleus（right） | 86 | -4.7423 | 36 | 3.8372 | - | - | - | - |
| The hippocampus  (left) | 128 | -5.3921 | 22 | 4.4728 | - | - | - | - |
| The hippocampus  (Right) | 135 | -4.5561 | 15 | 3.5569 | - | - | 21 | 3.5092 |
| Insular cortex (left) | 48 | -4.8513 | - | - | - | - | - | - |
| Insular cortex (right) | 9 | -4.2266 | 4 | 3.4315 | - | - | - | - |
| Entorhinal cortex (left) | 116 | -6.0434 | - | - | - | - | - | - |
| Entorhinal cortex (right) | 153 | -5.0178 | 79 | 4.1443 | - | - | - | - |
| Piriform cortex (left) | 70 | -4.5621 | - | - | 12 | 3.0826 | - | - |
| Piriform cortex (right) | 86 | -4.3524 | 5 | 3.565 | - | - | - | - |
| Sensory cortex (left) | 190 | -4.6074 | - | - | - | - | - | - |
| Sensory cortex (right) | 38 | -3.7028 | 12 | 3.6514 | - | - | - | - |
| Motor cortex (left) | 33 | -3.9738 | - | - | - | - | - | - |
| Motor cortex (right) | 21 | -4.3122 | - | - | - | - | - | - |
| Back of the cingulate  (left) | 12 | -3.2406 | - | - | - | - | - | - |
| Back of the cingulate  (Right) | 29 | -4.0625 | - | - | - | - | - | - |
| Dorsal thalamus (left) | 54 | -4.2115 | - | - | - | - | - | - |
| Dorsal thalamus (right) | 11 | -3.3011 | - | - | - | - | - | - |
| Auditory cortex (left) | 19 | -3.8058 | - | - | - | - | - | - |
| The striatum  (left) | 41 | -4.8903 | - | - | 21 | 4.0389 | - | - |

**figureS5 flowchart of the experimental design**

**Supplementary methods**

**1.1Participants**

During July 2014 to December 2016, 10 patients with post-stroke cognitive impairment (PSCI), and 10 patients affected by post-stroke non-cognitive impairment (PSNCI), matched with PSCI patients in terms of age, sex and years of education were selected from Fujian University of Traditional Chinese Medicine (FJTCM) affiliated Rehabilitation Hospital. The normal controls were sought from the community population in Fuzhou, matched with PSCI and PSNCI patients in terms of age, sex and years of education (n=10). FJTCM Subsidiary Rehabilitation Hospital ethics committees (approval number: 2013KY-005-01).

**1.2 Inclusion Criteria**

The inclusion criteria were as follows: (1) present stroke diagnosis was in accordance with the fourth national academic conference on cerebrovascular disease’s Diagnostic Criteria for All Kinds of Cerebrovascular Disease, and confirmed by computed tomography or magnetic resonance imaging; (2) PSCI patients with Mini-Mental Status Examination (MMSE) score ≤ 24; (3) PSNCI patients with MMSE score>24; (4) clinical diagnosis of first stroke incident within the preceding 12 months; (5) 45-75 years of age, junior high school education or above; and (6) Patient should be conscious, with stable vital signs. Among patients with PSCI was defined using interviews, neuropsychological battery and clinical dementia rating in accordance with the Diagnostic and Statistical Manual of Mental Disorders, Fourth Edition. Those who did not meet the criteria of post-stroke dementia were classified as PSCIND.[1].

**1.3 Exclusion Criteria**

Patients who (1) have awareness disorders, severe visual, hearing, and aphasia, and other health assessment failures; (2) have memory, and executive dysfunction caused by vascular dementia, Alzheimer's disease, hypothyroidism, brain trauma; (3) have comorbidity including tumors and severe heart, liver, kidney, hematopoietic system, and endocrine system diseases; (4) have psychiatric histories, such as personality disorders and schizophrenia; and (5) have cognitive impairment caused by depression, Baker Depression Scale (>10 points) will be excluded from the study.

**1.4 Neuropsychological Tests**

The MMSE was used to assess general cognitive function. The MMSE score ranges between 0 and 30. Participants were divided into three groups: Control group (MMSE source > 24), PSNCI group (MMSE source>24), and PSCI group (MMSE source≤24).[2].

**1.5** **Real-time quantitative PCR (RTq-PCR)**

Each group were processed for sera sample collection and the subsequent miR-219a detection. For the reverse transcription step, 2 µl of serum miRNA was converted to cDNA using the miRNA cDNA Synthesis Kit (Thermo Fisher Scientific) with standard procedures. The first strand cDNA was pre-amplified for 16 cycles and diluted 1:8 for the qPCR assays using the ABI Quant Studio 12 K Flex Open Array® real-time PCR instrument. We included a control RNA sample for each batch of reverse transcription in 96-well microplates for normalization of the qPCR assays during data analysis. [3].

**References:**

[1].	Wang, Z.Q., et al., MicroRNA Let-7i Is a Promising Serum Biomarker for Post-stroke Cognitive Impairment and Alleviated OGD-Induced Cell Damage in vitro by Regulating Bcl-2. Front Neurosci, 2020. 14: p. 215.

[2].	Prabhu, R.S., et al., Effect of the addition of chemotherapy to radiotherapy on cognitive function in patients with low-grade glioma: secondary analysis of RTOG 98-02. J Clin Oncol, 2014. 32(6): p. 535-41.

[3].	Lee, H.M., et al., Detection of increased serum miR-122-5p and miR-455-3p levels before the clinical diagnosis of liver cancer in people with type 2 diabetes. Sci Rep, 2021. 11(1): p. 23756.
